# A Computational Method to Propose Mutations in Enzymes Based on Structural Signature Variation (SSV)

**DOI:** 10.3390/ijms20020333

**Published:** 2019-01-15

**Authors:** Diego César Batista Mariano, Lucianna Helene Santos, Karina dos Santos Machado, Adriano Velasque Werhli, Leonardo Henrique França de Lima, Raquel Cardoso de Melo-Minardi

**Affiliations:** 1 Laboratório de Bioinformática e Sistemas (LBS), Department of Computer Science, Universidade Federal de Minas Gerais, 31270-901 Belo Horizonte, Brazil; luciannahss@gmail.com (L.H.S.); raquelcm@dcc.ufmg.br (R.C.d.M.-M.); 2 Laboratório de Biologia Computacional (COMBI-L). Centro de Ciências Computacionais-C3, Universidade Federal do Rio Grande, 96203-900 Rio Grande, Brazil; karinaecomp@gmail.com (K.d.S.M.); werhli@gmail.com (A.V.W.); 3 Laboratório de Modelagem Molecular e Bioinformática (LAMMB), Departamento de Ciências Exatas e Biológicas (DECEB). Universidade Federal de São João Del-Rei, Campus Sete Lagoas, 35701-970 Sete Lagoas, Brazil; leofrancalima@ufsj.edu.br

**Keywords:** enzymes, prediction of mutations, second-generation biofuel

## Abstract

With the use of genetic engineering, modified and sometimes more efficient enzymes can be created for different purposes, including industrial applications. However, building modified enzymes depends on several in vitro experiments, which may result in the process being expensive and time-consuming. Therefore, computational approaches could reduce costs and accelerate the discovery of new technological products. In this study, we present a method, called structural signature variation (SSV), to propose mutations for improving enzymes’ activity. SSV uses the structural signature variation between target enzymes and template enzymes (obtained from the literature) to determine if randomly suggested mutations may provide some benefit for an enzyme, such as improvement of catalytic activity, half-life, and thermostability, or resistance to inhibition. To evaluate SSV, we carried out a case study that suggested mutations in β-glucosidases: Essential enzymes used in biofuel production that suffer inhibition by their product. We collected 27 mutations described in the literature, and manually classified them as beneficial or not. SSV was able to classify the mutations with values of 0.89 and 0.92 for precision and specificity, respectively. Then, we used SSV to propose mutations for Bgl1B, a low-performance β-glucosidase. We detected 15 mutations that could be beneficial. Three of these mutations (H228C, H228T, and H228V) have been related in the literature to the mechanism of glucose tolerance and stimulation in GH1 β-glucosidase. Hence, SSV was capable of detecting promising mutations, already validated by in vitro experiments, that improved the inhibition resistance of a β-glucosidase and, consequently, its catalytic activity. SSV might be useful for the engineering of enzymes used in biofuel production or other industrial applications.

## 1. Introduction

Enzymes, in most cases, are proteins that accelerate biochemical reactions. They have applications in several fields of the industry, such as the production of drugs, food, beverage, biofuel, and so on [1,2]. Moreover, genetic engineering has been used to construct more efficient enzymes for industrial applications through mutations [3].

Techniques, such as error-prone PCR (epPCR), have been used to evaluate mutations systematically in several works. In this technique, a modified DNA polymerase inserts random mutations in the gene that codifies an enzyme during the replication process [4]. For instance, an epPCR library was used to identify three efficient mutations for an enzyme used in biofuel production. The combination of these mutations allowed the construction of a mutant enzyme that increased sugarcane bagasse conversion to fermentable sugars by 14–35% [3]. However, the proposal of modified enzymes depends on several in vitro and in vivo experiments, which may result in the process being expensive and time-consuming due to the vast number of possible mutations. For example, a protein with approximately 400 residues may present a total of 20^400^ residue combinations, which corresponds to 2.58 × 10^520^ possible mutations. From all possible mutations, experimental techniques can evaluate only hundreds of them. Therefore, a previous selection with a computational method may reduce costs and allow a higher number of tests, with promising mutated enzymes.

When comparing proteins, sequence alignment is the most traditional computational method. It identifies similar regions between proteins using substitution matrices [5]. For instance, an approach based on protein sequence activity relationships (ProSAR) uses sequences to predict the contributions of mutations on protein functions [6,7]. However, it does not consider the impact of the three-dimensional structure or the physicochemical proprieties of the mutated residues, which may be a limitation when suggesting mutations. Another approach to propose mutations is the evaluation of the variation of free energy of Gibbs difference (ΔΔ*G*) to analyze the thermostability of molecules. However, these computations are not feasible for all cases [8]. Hence, free energy calculations are not able to estimate with accuracy the impact of a mutation in an enzyme, the interaction with substrates and products, and the protein motion for more than a few examples. Hence, computational methods to propose and to evaluate mutations in enzymes at a large scale are still necessary.

Structural signatures, also called fingerprints, may be an alternative to analyze the impact of mutations as they provide a computationally feasible method to identify patterns of macromolecular structural features that may be important for structure and function. They have been successfully used in classification and automatic annotation of proteins [9,10], prediction of mutation effects on protein stability [11], prediction of the impact of mutations on the affinity between protein and ligands [12], and prediction of the mutation impact on the affinity between an antibody and antigen [13]. The aCSM (atomic Cutoff Scanning Matrix) method, based on structural signatures, calculates a structural signature, which is based on atomic pairwise distances, also considering their physicochemical properties [14]. It was also successfully used for the prediction of protein-ligand interactions. Hence, it may be used to characterize important regions that interact with the ligand.

In this paper, we propose a method based on structural signatures variation (SSV) to suggest mutations for improving the activity of enzymes. Our method can be applied to several types of enzymes. Despite the genericity of our method, we present a case study to demonstrate it and suggest mutations in β-glucosidase enzymes used in second-generation biofuel production. In addition, we carried out a comparative case study to analyze SSV performance to a similar structure-based approach called BioGPS [15].

## 2. Results

### 2.1. SSV Definition

**Figure 1:**
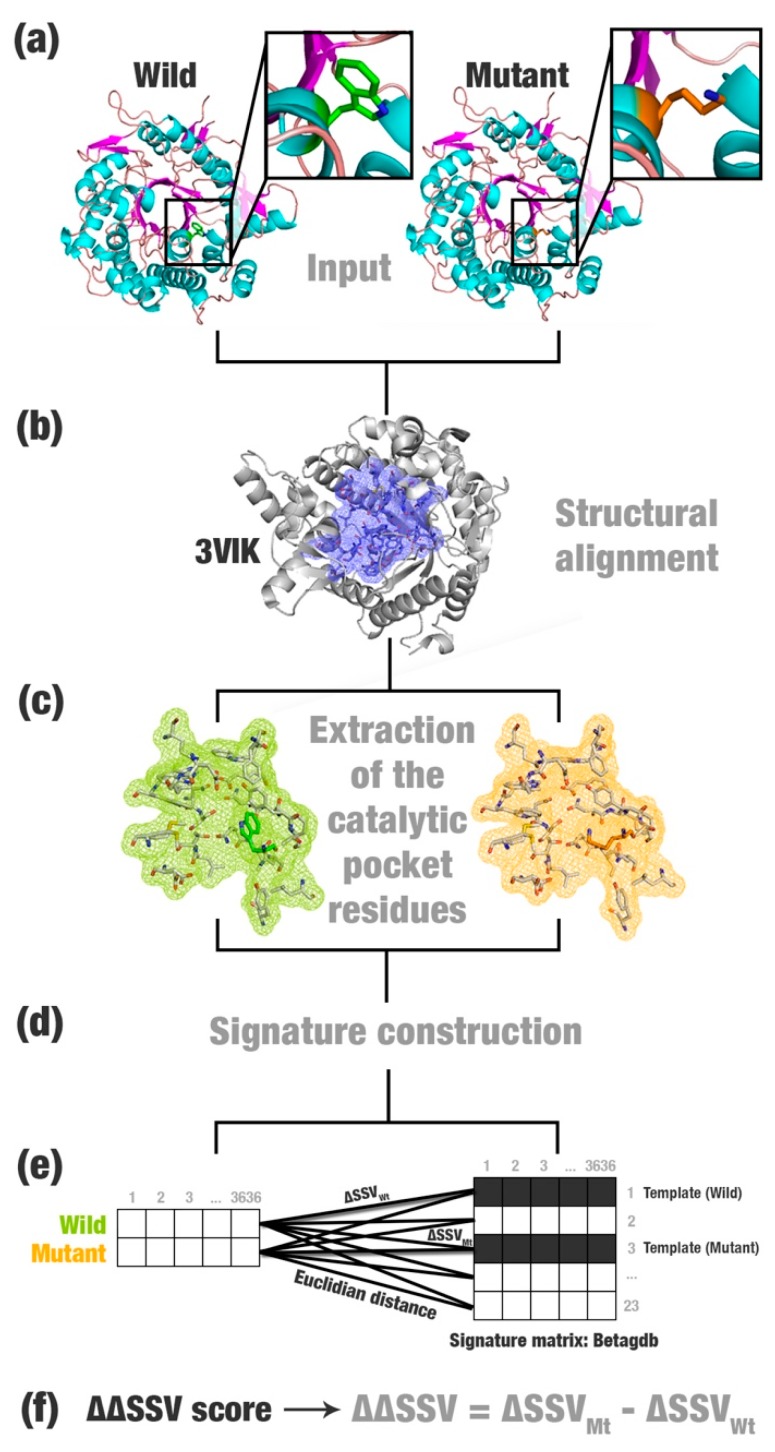
The structural signature variation (SSV) schema. (**a**) SSV receives as input PDB files containing mutant and wild protein (in this example, a β-glucosidase enzyme). (**b**) The structures are structurally superposed to a template (*Neotermes koshunensis* β-glucosidase; PDB ID: 3VIK). (**c**) The corresponding residues of the catalytic pocket in the wild, mutant, and their templates’ structures are extracted. (**d**) Structural signatures for all files are computed using the aCSM (atomic Cutoff Scanning Matrix) algorithm. (e) The Euclidean distance is computed for every line of the templates’ signature matrix. The lowest values define the templates for wild and mutant. (**f**) The difference between the two distances (ΔΔSSV) is calculated.

The structural signature variation (SSV) method is based on computing Euclidean distances between signatures of: (i) A wild enzyme and an enzyme model with the most similar signature to the wild type (called wild template); and (ii) a mutant enzyme and an enzyme model with the most similar signature to the mutant (called mutant template). The difference between the two distances (herein called the ΔΔSSV score) may be used to predict the impact of the mutation. The SSV method requires as input three-dimensional structures of a wild enzyme, a mutant enzyme (that can be modeled in silico), and enzyme models (herein called templates, i.e., proteins with positive characteristics that you want to transfer to other enzymes). SSV is computed using the following steps:

1. Most relevant residues’ extraction: For wild, mutated, and templates’ structures the most relevant residues are extracted and saved in a new Protein Data Bank (PDB) file (Figure 1a–c). This selection depends on the application and can be modified according to users’ needs. This step is optional.
2. Structural signature construction: For every PDB file, we compute a vector with the cumulative distribution of the pairwise distances among all pairs of atoms and their physicochemical proprieties (aCSM algorithm) (Figure 1d).
3. Template definition: A template definition depends on a high-curated database of enzymes with beneficial characteristics. This database should be manually and previously defined. We selected as a template, proteins with the closest signature to wild and mutant proteins analyzed (Figure 1e).
4. Comparison between signatures: A distance matrix among all signatures is constructed (a similar matrix is used to define the template). The Euclidean distance between two signatures is called signature variation (ΔSSV). The Euclidean distance between signatures of a wild enzyme and its template is called (ΔSSV_W*t*_). The Euclidean distance between signatures of a mutant enzyme and its template is called (ΔSSV_M*t*_). The difference between both values is the ΔΔSSV score. If the ΔΔSSV score is lower than zero, the mutant’s signature is more alike to the template signature than to the wild’s signature, suggesting that the mutation is beneficial. If the ΔΔSSV score is higher than zero, the mutant’s signature is more distant from the template signature than from wild’s signature, suggesting that the mutation is not beneficial (Figure 1f).

### 2.2. Case Study 1: Evaluating Mutations for β-Glucosidase Collected from the Literature

β-glucosidases (E.C. 3.2.1.21) are enzymes that perform the hydrolysis of glucosidic bonds, mainly in disaccharides [16,17]. They act in synergy with exoglucanases (E.C. 3.2.1.91) and endoglucanases (E.C. 3.2.1.4) in the second-generation biofuel production process. In the biomass degradation, endoglucanases attack the cellulose chain, releasing oligosaccharides of various lengths. Then, exoglucanases act, producing mainly cellobiose. β-glucosidases hydrolyze the cellobiose in two glucose molecules, which will be used in the fermentation process for ethanol production [18]. They have a key role in this process by removing the cellobiose, which is a potent inhibitor of exoglucanases and endoglucanases [19,20,21,22,23]. However, the majority of the known β-glucosidases has been described as being inhibited by high concentrations of glucose [24,25,26,27]. Hence, the production of β-glucosidases with a high tolerance for glucose inhibition may improve biofuel production [28].

To evaluate our method, we present a first case study for proposing mutations to improve the activity of β-glucosidase enzymes even in high glucose concentrations. We compared wild and mutant β-glucosidases with templates obtained in a manually curated database of glucose-tolerant β-glucosidases [29]. The database holds a group of β-glucosidases with high resistance to glucose inhibition and high industrial applications. However, few glucose-tolerant β-glucosidases have been described in the literature [30]. We hypothesized that glucose-tolerant and non-tolerant β-glucosidases have discriminant signatures. Hence, the signature of glucose-tolerant β-glucosidases previously characterized can be used to define if mutations in non-tolerant β-glucosidases make their signature similar to a tolerant β-glucosidase or not.

#### 2.2.1 Data Collection and Manual Classification of Mutation Effects

We collected 27 mutations in β-glucosidases from the literature and the UniProt database (https://uniprot.org) (Table 1). Every mutation was manually classified as beneficial or not according to the impact description in the β-glucosidase activity. We classified as “beneficial” mutations that tend to improve the saccharification process, such as mutations reported as responsible for improving the glucose tolerance, increasing optimal temperature, increasing the catalytic efficiency, reducing the affinity for the product, or improving the affinity for the substrate. On the other hand, we classified as “not beneficial” mutations that tend to reduce the saccharification process, such as mutations reported as responsible for decreasing the affinity for the substrate, increasing the affinity for the product, or reducing the catalytic activity. For example, the mutation, H228T, in the β-glucosidase, Bgl1B, has been described as responsible for improving the glucose tolerance [27]. Hence, we classified it as beneficial. On the other hand, the mutation, V168Y, in the human cytosolic β-glucosidase has been described as responsible for reducing the specific activity [31]. Hence, we classified it as not beneficial.

#### 2.2.2. Predicting the Impact of Mutations

We performed the SSV method (Figure 1), evaluated the ΔΔSSV score for the 27 mutations in β-glucosidases, and compared them to the expected results. For mutations classified as beneficial, we expected a negative ΔΔSSV score; and for mutations classified as not beneficial, in turn, a positive ΔΔSSV score.

SSV predicted correctly eight in a total of nine beneficial mutations (Table 2). For the non-beneficial mutations, where the expected ΔΔSSV was higher than zero, SSV predicted correctly 12 out of 18.

#### 2.2.3. Comparison with Other Methods

We compared our method to the support vector machine (SVM) implemented on the Weka (Waikato Environment for Knowledge Analysis) tool [42]. SVM is a learning algorithm for classification. We performed four experiments: (i) SSV; (ii) SVM using as input only wild signatures; (iii) SVM using as input only mutant signatures; and (iv) SVM using as input the difference of the wild vector and mutant vector. For these experiments, we evaluated the following metrics: Precision, accuracy, specificity, sensibility, and the F-measure [43].

We observed that the precision and specificity of SSV were superior to the other method. SSV obtained a precision of 0.89 and a specificity of 0.92 (Table 3). It also performed better in the prediction of beneficial mutations than the SVM.

### 2.3. Case Study 2: Proposing Mutations for a Non-Tolerant β-Glucosidase

In the second case study, we described a real application for the method SSV. We chose a non-tolerant β-glucosidase, Bgl1B (UniProt accession number: D0VEC8), to suggest mutations using SSV. Bgl1B was extracted from a marine metagenome and presented the half maximal inhibitory concentration (IC_50_) of 50 mM for glucose [44]. For comparison, Bgl1A, a glucose-tolerant β-glucosidase also extracted from a marine metagenome, presented IC_50_ of 1000 mM [45]. In a recent study, several mutations for improving the activity in higher glucose concentrations were proposed for Bgl1B [27]. This study will be used to compare the results of the mutations proposed by the SSV method.

**Figure 2:**
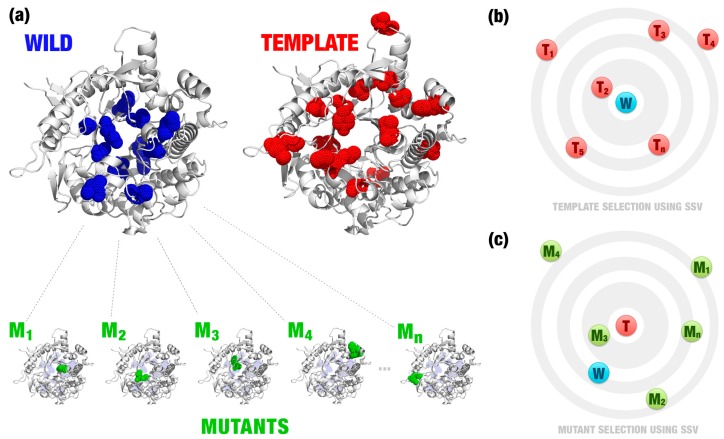
(**a**) Wild and template have a similar folding, but differences in the sequence (illustrated by blue dots in the wild enzyme and by red dots in the template enzyme). Several point mutations were proposed for the wild enzyme (green dots). The template enzyme is defined based on a curated database of enzymes with desired characteristics (in this case study, Betagdb). For instance, in (**b**) the template, T2 was defined as the template (T) for the wild enzyme (W). SSV is illustrated by a two-dimensional visualization in (**b**) and (**c**). Euclidean distances between signatures of the wild/mutants and the template (signature variation) are used to define the best template (**b**) and mutant (**c**). In this example, the mutant, M3, was defined as the mutation that best inserts characteristics similar to the template (**c**). Images generated using PyMOL software (http://pymol.org).

We modeled point mutations by homology for all residues of the catalytic pocket (composed by 22 residues around the active site). For each residue, 19 mutations were proposed, in a total of 418 mutants (Figure 2a). Then, we defined the template with the most similar signature (Figure 2b). Also, we used this template to evaluate the mutant that inserts more similar characteristics to the template (Figure 2c). Note that, in this example, the wild and template have a similar folding, but different sequences (Figure 2a). Wild (Bgl1B) and template (Bgl1A) have an identity of 55% (243 similar residues in a total of 443). Thus, it is necessary to evaluate hundreds of mutations to detect beneficial mutations using simply sequence alignment. SSV takes into consideration the changes in the protein environment, for example, changes in the residues volume, atoms distances, and their pharmacophoric proprieties.

After running SSV, we detected 86 mutations with negative ΔΔSSV (available in the Supplementary File). In a real application, this could still be a high value of mutations for a bench test. Hence, we proposed additional steps to limit the number of promising mutations (a detailed description is available in the Section 4). We removed nine mutations that occurred in the residues, H125, N169, E170, Y298, E353, and W399, because they were conserved in 100% of glucose-tolerant β-glucosidases. We also removed 58 mutations indicated as being not allowed in the GH1 family by the SIFT (Sorting Intolerant From Tolerant) software [46]. SIFT uses the physical properties of amino acids and sequence homology to predict the effect of an amino acid substitution on the protein function. Then, we analyzed the mutation impact in the structure using mCSM (mutation Cutoff Scanning Matrix) [11]. The mCSM software uses graph-based signatures to predict the effect of mutations in proteins. For 19 remaining mutations, mCSM considered four as highly destabilizing. In the end, 15 mutations were proposed for Bgl1B (Table 4). These mutations affect five residues: F172 (three mutations), G246 (two mutations), H228 (eight mutations), T299 (one mutation), and V227 (one mutation).

Experimental data is available in the literature for three proposed mutations: H228C, H228T, and H228V [27]. These single-point mutants keep the relative activity even in higher glucose concentrations than wild Bgl1B. This suggests that the SSV method can be promising to propose beneficial mutations for β-glucosidases.

### 2.4. Case Study 3: Comparing to BioGPS Descriptors

In this case study, we compared SSV to the analysis performed in the BioGPS study [15]. BioGPS is a bioinformatics methodology for rational engineering of enzyme promiscuity that uses chemical, geometrical, and physical-chemical features of three-dimensional structures. BioGPS compares actives sites’ properties, taking into consideration more than the sequence structure. Therefore, we considered a similar approach to SSV.

In the BioGPS study, eight mutants experimentally evaluated (Table 5) for a lipase B from *Candida antarctica* (CaLB) were used to validate the method [47]. CaLB is a stable lipase that belongs to the serine-hydrolases super-family. The insertion of amidase activity in CaLB has many applications for the industry [15,47,48]. BioGPS classified the mutations based on the improvement factor (IF) referred to CaLB wild-type activity. The IF is equal to the amidase activity of the mutant, divided by the amidase activity of the CaLB wild [15]. We considered IF > 1 as beneficial mutations, and IF < 1 as not beneficial mutations (Table 5). Also, SSV considered the mutant, M8, as a possible neutral mutation for presenting an IF slightly over 1.

We collected the residues presented in the region near the catalytic triad and ran SSV using M3 as the template (see the Section 4 for details). For the seven mutations validated by BioGPS, SSV correctly predicted five (M3 was tested as a control experiment and should not be considered in the calculation of accuracy). However, this case study could present some biases that will be discussed in the next section.

## 3. Discussion

We hypothesized that the more similar the signature of a β-glucosidase is to another β-glucosidase, classified as tolerant, the more they will preserve common characteristics. Hence, if a mutation turns the signature of a β-glucosidase more similar to the signature of a glucose-tolerant β-glucosidase, it might show comparable characteristics for biofuel production. The same could be inferred if the method was applied to another enzyme.

To validate our method, we collected 27 mutations from the literature, manually classified as beneficial or not, submitted it to three other methods, and compared it with the expected ΔΔSSV score. We highlighted that our method does not have a direct competitor or another method that does exactly the same thing. Thus, three alternative methods based on SVM are proposed, which is the state of the art in machine learning for comparison. We attained 0.89 and 0.92 for the precision and specificity, respectively (Table 3). Precision is an appropriate metric to evaluate this case study as it emphasizes hits in beneficial mutations. This value of precision indicated that out of the nine beneficial mutations for β-glucosidases, SSV predicted eight correctly. The results showed that the Euclidean distance, implemented by SSV, achieved better results in the beneficial impact of mutation prediction than SVM (specificity and precision). However, SSV is not directly comparable to SVM. SSV is a simple strategy to model and compare the impact of mutations based on efficient proteins for a pre-established activity detected in nature. It uses the Euclidean distance to construct a score that will be used to compare structural signatures. SVM is a learning algorithm for supervised classification. In the case study, SVM received as input the structural signature matrix calculated by a step of the SSV method. We comprehend that this is not a straightforward comparison, but our intention is to demonstrate that our method is capable of classifying beneficial mutations correctly and achieves better results than using a model based on an SVM classifier.

### 3.1. Improving the Activity of a Non-Tolerant β-Glucosidase

**Figure 3:**
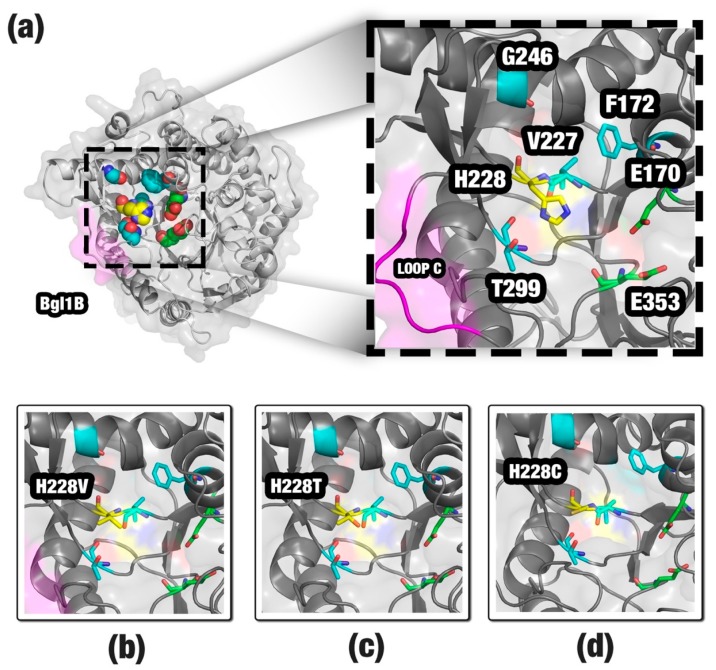
Structure of Bgl1B (**a**), sites pointed by SSV as a target for mutations: H228 residue (yellow); F172, V227, G246, and T229 (cyan). The catalytic residues (E170 and E353) are shown in green. Additionally, loop C is in magenta. For comparison, we highlighted the mutations, H228V (**b**), H228T (**c**) and H228C (**d**), considered by the literature as being responsible for glucose tolerance. Images generated using PyMOL software (http://pymol.org).

A total of 15 mutations was proposed for improvement in the activity of Bgl1B (Figure 3a). The principal mutation site appeared to be the H228 residue. Our method proposed eight mutations for this site. Also, we found experimental data for three of these mutations: H228C, H228T, and H228V (Figure 3b–d). These mutations showed an activity improvement of Bgl1B even in higher glucose concentrations. Histidine is an amino acid classified as positively charged and bulky. The substitution of a histidine by an amino acid of a shorter side chain, such as cysteine, threonine, or valine, would provide a space that could allow a better allocation for glucose, agreeing with the study of Yang et al. [27]. Most of the other mutations proposed for H228 by SSV also provide a reduction in the side chain. Hence, we suggest that they could provide the same effect. The F172, G246, T299, and V227 residues are in the neighborhood of H228 (Figure 3a). We suppose that mutations in these sites could affect the exit pathway of the glucose from the active site. Also, these sites are near the loop C, a region in the entrance of the channel that guides to the active site. The geometrical differences around the loop C were described by Fang et al. [45] as being probably responsible for the characteristic of the glucose tolerance in β-glucosidase enzymes (Figure 3a). Taken together, the SSV results might indicate that our method was able to find some of the same beneficial mutations obtained by in vitro experiments and propose new ones to be tested.

### 3.2. Evaluating Mutations in CaLB

Using structural bioinformatics strategies for proposing mutations appears to complement sequence strategies. In general, methods based on sequences present a lower computational cost, such as the one implemented in ProSAR [6]. However, SSV is a method based on structural comparisons, with low computational costs. Other tools, models, and algorithms have been reported to use three dimensional structures with similar approaches to SSV to propose mutations, such as the active site constellations method [49], where distances between functional groups of the protein active site and the substrate are calculated and used as the template in a search for matches in structural databases, and BioGPS descriptors [15].

We analyzed eight mutations assessed in the BioGPS study using SSV. To construct our case study, we performed some modifications in the methodology. Ferrari et al. [15] used a database composed of 42 serine-hydrolases to construct the BioGPS fingerprint. However, the selection was performed according to their annotated E.C. number, which is a target of debate among the enzymologist community due to the lack of quality control. Despite the dubious quality, the authors considered the database as consistent to their research. However, SSV requires high-accuracy databases of templates. Hence, we used the M3 mutant as a unique template. M3 was the mutant that inserted the highest value in the improvement factor in CaLB.

In addition, we evaluated the M3 mutant using the same file as the template for producing a control experiment. Indeed, the negative ΔΔSSV value for the M3 mutation demonstrates that SSV correctly predicted the structural similarities between the mutant and template (Table 5).

From the SSV results, we could infer that W104F appear to be the most important mutation for improving the activity of CaLB. Although G39A presents some improvements in CaLB activity according to BioGPS, SSV was not able to detect the improvement. We can hypothesize that the substitution of a glycine by an alanine, the change of a hydrogen by a CH_3_ group, is not sufficient to perform large modifications in the cumulative distribution of pairwise atoms calculated by aCSM. However, the substitution of a glycine could affect the mobility of secondary structures in the region, which would be detected using high-cost computation strategies, such as molecular dynamics. Indeed, the authors of BioGPS used 500 ns of molecular dynamics using the software, GROMACS [50], to construct and evaluate the mutants’ fingerprints. Molecular dynamics have a high computational cost, and their use could make the assessment of mutations on a large scale not feasible.

Interestingly, the T103G mutation (found in M8) occurs in a region distant to the active site. For this reason, SSV predicted a neutral impact in the activity. Indeed, T103G proposed a slight improvement in the mutant activity (IF: 1.1), hence we consider this prediction correct.

The SSV mistake for mutant M6 could be related to the small number of elements in the template database. SSV depends on enzymes with efficient catalytic activities previously reported to be used as templates. For the β-glucosidase case study, we previously performed a systematic literature review, collected several mutations that were beneficial and not beneficial, and constructed a highly accurate database (Betagdb). However, a systematic literature review demands great effort, and the necessity to perform this kind of the previous study to construct a template database may be a negative point of the SSV approach.

Lastly, SSV presents a user-friendly interface, which could be easily run by users. Therefore, it could be used together with other strategies, such as BioGPS, ProSAR, or active site constellations, to aid in the proposition of more efficient mutations before performing in vitro experiments.

### 3.3. Important Issues before Using SSV

The use of SSV may present some drawbacks. First, the method depends on three-dimensional structure models to determine the structural signature. Models are obtained by computational heuristics and, for this reason, they can present differences to structures obtained by experimental methods, such as X-ray crystallography. However, achieving structures by experimental methods may be time consuming and expensive. Also, to propose mutations, SSV depends on templates with favorable characteristics, for example, mutations described in the literature, which are responsible for improvements in thermostability or catalytic activity, which may be hard to find.

SSV uses structural signature variations to detect patterns in enzymes with appropriate industrial applications and transfer them, testing random point mutations, to other enzymes that do not present similar behavior.

A final difficulty is a need for a curated database with positive and negative examples. In this work, we presented a case study, where we used a database obtained by a systematic literature review. Reviews like that take a long time to prepare and they are expensive. The SSV method may be reproduced using the three basic inputs: (i) A wild enzyme; (ii) a mutant of this enzyme; (iii) a template enzyme with positive characteristics for some industrial application that you desire to transmit for the mutant. Furthermore, we believe that in real scenarios, researchers involved in protein engineer processes should know interesting positive and negative examples to use as templates.

## 4. Materials and Methods

### 4.1. Method Description

#### 4.1.1 Extraction of the Catalytic Pocket

The residues of the catalytic pocket were collected from every β-glucosidase structure (Figure 1a). The catalytic pocket consists in the channel region that guides to the active site. This channel has been described as being responsible for the characteristic of glucose tolerance for β-glucosidases [51].

We extracted the residues up to 6.5 Å of the ligand using in-house scripts. This distance was selected based on a cutoff to characterize pockets for the structural signature [14]. Pires et al. [14] performed tests with 35,000 pockets to define how far from the ligand are the most important residues to construct a representative signature. They observed that all signature methods of aCSM present high *p*-values cutoff between 6.0 Å and 7.0 Å. Thus, they concluded that 6.0 Å was the best atomic cutoff for the pocket definition for their classification system. We extended the distance to 6.5 Å to include the corresponding residues to TRP169, an important amino acid for the glucose tolerance of β-glucosidases described in some studies [51].

We used, as a reference, the β-glucosidase in complex with cellobiose extracted from the termite, *Neotermes koshunensis* (PDB ID: 3VIK; [52]; Figure 1b). The residues of the 3VIK catalytic pocket are Q45, H148, W149, N192, S193, L195, T196, D199, M207, N253, I254, N255, Y273, N335, F336, Y337, T338, L340, W374, E402, W444, E451, W452, and F460. Then, we performed structural alignments between 3VIK and the β-glucosidases evaluated using the MultiProt tool [53] and selected the corresponding residues (Figure 1c). Optionally, the entire protein could be used in this step. However, we believe that calculating the signature of a specific region could improve the results.

#### 4.1.2. Structural Signature Construction

Structural signatures were constructed using aCSM [14]. The aCSM tool (UFMG, Belo Horizonte, Brazil) creates graph-based signatures to describe proteins. We used the version, aCSM-ALL, that also includes the pharmacophore classes: Hydrophobic, positively charged, negatively charged, hydrogen acceptor, hydrogen donor, aromatic, sulfur, and neutral. For each protein, aCSM-ALL calculates the pairwise distances among all pairs of atoms and constructs a distance matrix with the cumulative distribution. We used the cutoff range of 0 to 10 Å, and the cutoff step of 0.1 Å. For each protein, aCSM-ALL returns a vector with 3636 columns. The vector represents a unique structural signature, which may be used to identify the protein or compare it with other similar proteins.

In the aCSM-ALL matrix, the lines represent the protein, and the columns represent the cumulative distribution of pairwise atoms. Hence, for a cutoff of 0–10 Å and a step of 0.1 Å, aCSM-ALL calculates the number of atom pairs at cutoff distances of 0 to 0.1 Å, 0.1 to 0.2 Å, 0.2 to 0.3 Å, (…), 9.8 to 9.9 Å, and 9.9 to 10 Å. For example, a protein could present 100, 200, 50, 300, and 20 pairs of hydrophobic residues at cutoff distances of 2.0 to 2.1 Å, 3.0 to 3.1 Å, 5.3 to 5.4 Å, 7.4 to 7.5 Å, and 9.7 to 9.8 Å, respectively. All these numbers and other cutoffs were included in the matrix. Also, aCSM-ALL verified some combinations of residues, for instance, how many atom pairs of positively charged and negatively charged there were for all cutoffs’ values. For this reason, each line of the aCSM-ALL matrix presented 3636 columns.

#### 4.1.3. Template Definition

Templates are a three-dimensional structure of glucose-tolerant β-glucosidases that are used as models by SSV to define if mutations are beneficial or not. SSV depends on good templates to perform comparisons between signatures. Templates should be empirically selected based on the literature information.

We collected 23 PDB files of glucose-tolerant β-glucosidases from Betagdb (a list is available in the Supplementary File). Betagdb (http://bioinfo.dcc.ufmg.br/betagdb) is a database that contains structures of β-glucosidases with high efficiency for biofuel production collected from a systematic literature review [29]. We previously calculated the structural signature of every glucose-tolerant β-glucosidase using the same parameters for wild and mutant signatures and stored it in the Betagdb signature matrix. We used the Euclidean distance to calculate the signature variation for each wild (ΔSSV_W*t*_) and mutant (ΔSSV_M*t*_) protein. The lowest value for the distance defines the template (Figure 1e). Wild and mutant β-glucosidases may have the same template or different templates.

#### 4.1.4. Comparison between Signatures

**Figure 4:**
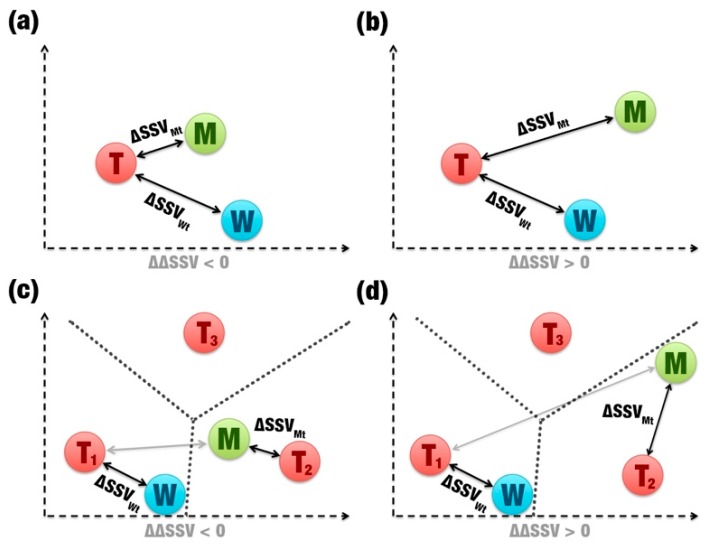
Two-dimensional representation of comparisons between signatures. (**a**) and (**b**) show simple comparisons (same template). (**c**) and (**d**) show comparisons with two different templates (T_1_, T_2_, and T_3_). The gray arrows highlight that a second template was used. The dotted lines are used to show whether a mutation becomes more similar to a second template than the original. (**a**) and (**c**) represent beneficial mutations. (**b**) and (**d**) represent non-beneficial mutations.

The ΔΔSSV score is calculated from the comparison between signature variations (Figure 1f). This score is binary: If it is positive, the mutation is not beneficial (Figure 4b,d); if it is negative, the mutation is beneficial (Figure 4a,c). When wild and mutants have the same template (Figure 4a,b), SSV performs a simple distance comparison between the Euclidean distances of wild’s and mutant’s signatures to the template’s signature. However, if a mutation causes a large change in the β-glucosidase signature, the mutant can show greater similarity in its signature to a second template (Figure 4c,d). The ΔΔSSV is calculated using the difference of the distance variation for the mutant and the second template by the distance variation for the wild and the first template. In this case, the change in the signature is significant, which should indicate that the mutation is not beneficial. However, a significant signature change also can indicate that the mutant’s signature is closer to another template. Therefore, high impacting mutations also may be beneficial (Figure 4c).

### 4.2. Case Study 1

We collected 27 mutations for β-glucosidases in the literature (Table S1), applied the calculations of signature variations, and evaluated the method’s precision, accuracy, specificity, sensibility, and F-measure. Sequences were collected in the databases, GenBank (http://www.ncbi.nlm.nih.gov/genbank) and UniProt (http://www.uniprot.org). Three-dimensional structures were collected in the Protein Data Bank (PDB) [54]. The sequences without available three-dimensional structures were modeled by homology [55]. We selected the templates for modeling using the NCBI BLAST web interface [56] and built 100 models for each protein using MODELLER [57,58,59]. The best models were selected using the DOPE score. Mutations were modeled using the script for point mutations from MODELLER. For each of the 27 mutations, we extracted the catalytic pockets using in-house scripts and constructed the structural signature. Then, we determined the templates and calculated the ΔΔSSV score.

### 4.3. Case Study 2

The sequence of Bgl1B was obtained in UniProt (accession number: D0VEC8). We constructed 100 models using MODELLER. We used as a model the GH1 β-glucosidase from *Exiguobacterium antarcticum* B7 (PDB ID: 5DT5; coverage: 96%; and identity: 44%). We selected the best model using the DOPE score [57,58,59]. Point mutations were performed in the residues of Bgl1B’s catalytic pocket. Each one of the 22 residues was mutated according to 19 possibilities using MODELLER’s mutation script, resulting in 418 mutant proteins. We aligned the PDB files with the β-glucosidase in the complex with cellobiose (3VIK) and extracted the residues of the catalytic pocket based on residues established previously. We generated the structural signatures for all files and calculated the ΔΔSSV score (Table S2).

In addition, we proposed additional steps to limit the number of mutations proposed. We removed mutations proposed based on three evaluations: (i) Mutations in conserved residues; (ii) residues that are not found in a specific position in the family; and (iii) mutations that potentially cause high destabilization in the protein structure.

Residue conservation is an important metric used to evaluate mutations. Highly conserved residues tend to present essential functions for the protein activity. We performed sequence alignment of catalytic pocket residues among Bgl1B and the β-glucosidases of Betagdb using Clustal Omega [60,61]. We detected six conserved residues: H125, N169, E170, Y298, E353, and W399. We removed mutations in these residues indicated by SSV.

Then, we used the SIFT Sequence [46] to analyze the substitution allowed in the GH1 family for every residue of the catalytic pocket (Table S3). We removed mutations not detected in that position for the GH1 family.

Mutations can affect the protein structure, causing a destabilization that may compromise the protein activity. We evaluated the impact of mutations in the protein structure using mCSM (FIOCRUZ MINAS, Belo Horizonte, Brazil), which predicts the variation of free energy (ΔΔG) [11]. Indeed, most of the mutations cause destabilization; however, some can cause high destabilization, which may change the protein folding state. We removed the mutations indicated by mCSM as highly destabilizing (Table S4). The remaining mutants were the final mutations proposed by our workflow for tests in vitro. Lastly, we compared the results with the mutations tested experimentally in the literature.

### 4.4. Case Study 3

The three-dimensional structure of CaLB was obtained from the PDB (PDB ID: 1TCA). The mutants from M1 to M8 were constructed using the mutagenesis tool of the software, PyMOL (http://pymol.org). Water molecules were removed. To detect the residues of the pocket near the active site, we performed molecular docking in the wild-type and mutants using the software, AutoDock Vina (The Scripps Research Institute, La Jolla, CA, USA) [62]. We used *N*-benzyl-2-chloroacetamide, the same ligand used to determine amidase activities in CaLB [47]. The ligand was collected from the Zinc database [63]. We used parameter exhaustiveness = 50, a box of 15 Å × 15 Å × 15 Å, and the box center was defined based on the position of the last atom of the catalytic serine (residue S105; atom OG). We used the first conformation obtained by docking and collected all residues at the distance of 6.5 Å from any atom of the ligand. Then, we removed the ligand and saved the structures as PDB files. We performed tests in the SSV web tool using the wild-type, the eight mutants, and the template database (for this step, we compressed the mutant, M3, in a zip file). The links for the projects created in the SSV tool are available in the Supplementary Material (Table S5; Figure S1).

## 5. Conclusions

In this paper, we proposed structural signature variation (SSV), which is a novel method to compute and compare structural and physicochemical signatures of proteins, with the purpose of proposing beneficial mutations to support protein engineering processes. SSV can be used together with other methods, tools, and algorithms to suggest mutations with greater reliability for reducing costs of in vitro experiments.

We evaluated the quality of the predictions through two case studies with realistic examples for the protein engineering of β-glucosidases, enzymes involved in biofuel production. SSV presented a high precision for 27 mutations collected from the literature and was capable of detecting beneficial mutations already proposed in the literature for Bgl1B, starting from random point mutations. SSV was shown to be an efficient method to propose mutations for non-tolerant β-glucosidases and may help yield enzymes with more glucose tolerance for second-generation biofuel production.

In addition, we constructed a website, with a user-friendly interface, that implements the SSV method. It is available at (http://bioinfo.dcc.ufmg.br/ssv).

## Figures and Tables

**Table 1:** Mutations collected from the literature and UniProt.

ID	Mutation	Effect	Classification	Source
1	H228T	Improves glucose tolerance.	Beneficial	[27]
2	V174C/A404V/L441F	Increases the optimal temperature of 50 °C to 60 °C, reduces the optimal pH of 6 to 5.5.	Beneficial	[3]
3	H184F	Increases the inhibition constant for glucose.	Beneficial	[32]
4	P172L	Increases catalytic efficiency.	Beneficial	[33]
5	P172L/F250A	Increases catalytic efficiency.	Beneficial	[33]
6	L167W	Increases the optimal temperature and glucose tolerance.	Beneficial	[33]
7	L167W/P172L	Increases the activity (2×).	Beneficial	[34]
8	L167W/P172L/P338F	Increases the activity (1,3×).	Beneficial	[34]
9	V168Y	Reduction in the specific activity.	Not beneficial	[31]
10	F225S	Reduction in the specific activity.	Not beneficial	[31]
11	Y308F	Reduction in the specific activity.	Not beneficial	[31]
12	Y308A	Reduction in the specific activity.	Not beneficial	[31]
13	I207V	Increases the specificity constant (K_cat_/K*_m_*).	Beneficial	[35]
14	N218H	Decreases the K*_m_* about 2-fold.	Beneficial	[36]
15	N273V	Increases the K*_m_* about 5-fold.	Not beneficial	[36]
16	F252I	Reduces substrate affinity.	Not beneficial	[37]
17	F252W	Reduces substrate affinity.	Not beneficial	[37]
18	F252Y	Reduces substrate affinity.	Not beneficial	[37]
19	M284N	Reduction of K_cat_/K*_m_* 7 to 30-fold depending on the substrate.	Not beneficial	[35]
20	H276M	Reduction of K_cat_/K*_m_* 2 to 6-fold depending on the substrate.	Not beneficial	[38]
21	V173C	Decreases affinity for cellobiose.	Not beneficial	[39]
22	M177L	Decreases affinity for cellobiose (small reduction).	Not beneficial	[39]
23	D229N	Decreases affinity for cellobiose (high reduction).	Not beneficial	[39]
24	H231D	Decreases affinity for cellobiose.	Not beneficial	[39]
25	E96K	Improves the thermostability.	Beneficial	[40]
26	N223G	Reduction of transglycosylation, glucose tolerance, and activity.	Not beneficial	[41]
27	N223Q	Reduction of transglycosylation, glucose tolerance, and activity.	Not beneficial	[41]

**Table 2:** ΔΔSSV score expected and the value predicted by SSV.

ID	Mutation	ΔΔSSV Expected	ΔΔSSV Score	Hit
1	H228T	ΔΔSSV < 0	−186.18	✓
2	V174C/A404V/L441F	ΔΔSSV < 0	−246.22	✓
3	H184F	ΔΔSSV < 0	100.37	
4	P172L	ΔΔSSV < 0	−6.29	✓
5	P172L/F250A	ΔΔSSV < 0	−6.29	✓
6	L167W	ΔΔSSV < 0	−602.80	✓
7	L167W/P172L	ΔΔSSV < 0	−615.46	✓
8	L167W/P172L/P338F	ΔΔSSV < 0	−615.46	✓
9	V168Y	ΔΔSSV > 0	330.56	✓
10	F225S	ΔΔSSV > 0	−365.07	
11	Y308F	ΔΔSSV > 0	34.19	✓
12	Y308A	ΔΔSSV > 0	−108.62	
13	I207V	ΔΔSSV < 0	−71.56	✓
14	N218H	ΔΔSSV < 0	−230.61	✓
15	N273V	ΔΔSSV > 0	−55.26	
16	F252I	ΔΔSSV > 0	86.70	✓
17	F252W	ΔΔSSV > 0	129.97	✓
18	F252Y	ΔΔSSV > 0	37.86	✓
19	M284N	ΔΔSSV > 0	−127.35	
20	H276M	ΔΔSSV > 0	−501.32	
21	V173C	ΔΔSSV > 0	13.59	✓
22	M177L	ΔΔSSV > 0	20.86	✓
23	D229N	ΔΔSSV > 0	18.11	✓
24	H231D	ΔΔSSV > 0	−54.22	
25	E96K	ΔΔSSV < 0	−31.08	✓
26	N223G	ΔΔSSV > 0	39.37	✓
27	N223Q	ΔΔSSV > 0	264.34	✓

**Table 3:** Metrics used to evaluate SSV.

Metric	SSV	SVM (Wild)	SVM (Mutant)	SVM (Wild-Mutant)
Precision	0.89	0.64	0.36	0.36
Accuracy	0.74	0.81	0.74	0.74
Specificity	0.92	0.79	0.70	0.70
Sensitivity	0.57	0.88	1.00	1.00
F-measure	0.70	0.74	0.53	0.53

**Table 4:** Mutations proposed by the SSV method for the non-tolerant β-glucosidase, Bgl1B. Mutations underlined and in bold were found in the literature.

F172	G246	H228	T299	V227
F172I	G246S	H228A	T299S	V227M
F172K	G246T	**H228C**		
F172V		H228M		
		H228N		
		H228P		
		H228Q		
		**H228T**		
		**H228V**		

**Table 5:** CaLB’s mutants evaluated by SSV for comparison to BioGPS. These values were obtained from references [15,47,48].

Mutant	Mutation	IF	Classification	ΔΔSSV	Hit
M1	G39A/W104F/L278A	6.3	Beneficial	−841	✓
M2	G39A/T103G/L278A	3.8	Beneficial	−121	✓
M3	G39A/T103G/W104F/L278A	11.2	Beneficial	−841	✓
M4	G39A	2.8	Beneficial	150	
M5	G39A/L278A	3.3	Beneficial	−121	✓
M6	I189A	0.4	Not beneficial	−94	
M7	T40A	0.4	Not beneficial	40	✓
M8	T103G	1.1	Neutral/Beneficial	0	✓

## Supplementary Materials

Supplementary materials can be found at http://www.mdpi.com/1422-0067/20/2/333/s1.

## Abbreviations

CaLB	*Candida antarctica* lipase B
epPCR	Error-prone PCR
GH1	Glycoside hydrolase family 1
IF	Improvement Factor
SSV	Structural Signature Variation
SVM	Support Vector Machine
