# Supplementary material for: A Computational Method to Propose Mutations in Enzymes Based on Structural Signature Variation (SSV)
Source: Int J Mol Sci. 2019 Jan 15;20(2):333. doi: 10.3390/ijms20020333 (PMC6359350; doi:10.3390/ijms20020333)
Supplement: Supplementary file 1 [file ijms-20-00333-s001.pdf]

International Journal of
Molecular Sciences

MDPI

Supplementary file 1. Using SSV by the web application

We developed a user-friendly web implementation of SSV at <http://bioinfo.dcc.ufmg.br/ssv>. To perform SSV calculations, access the website using a browser (we recommend Google Chrome), click on "Run online" or in the "Run now!" Button.

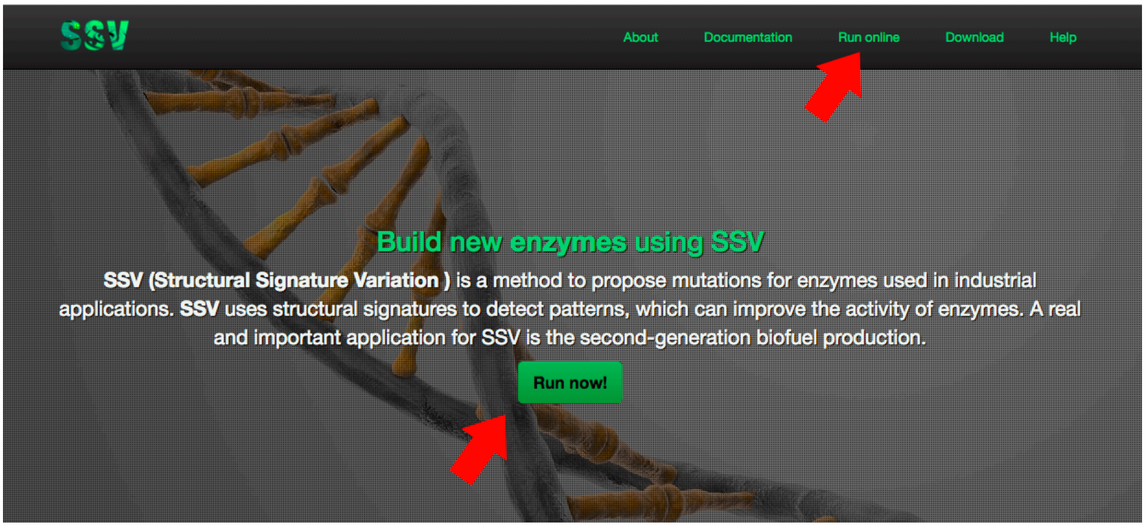

The "Run online" panel requires some information to execute:

- **Project name**: define a name for your project. You can use any name (the system will create a unique ID for each project);
- **E-mail**: declare your e-mail (optional);
- **Mutation evaluated**: insert the point mutation or multiple mutations evaluated (optional);
- **Wild PDB:** input the wild PDB file;
- **Mutant PDB:** input the mutant PDB file;
- **Templates database:** input a set of proteins that it will be used as templates (in a zip file).

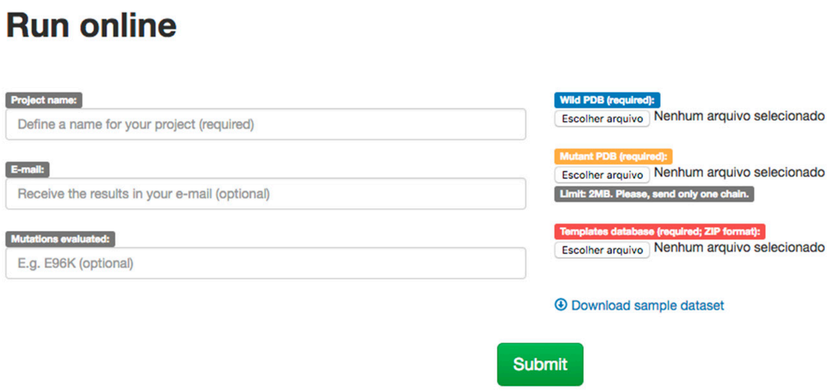

There is available a sample database with 27 mutations evaluated in the paper and the 23 proteins of Betagdb (the templates for the case study). Users can download the database and perform analysis using SSV online. There is also a table with expected values and the values calculated.

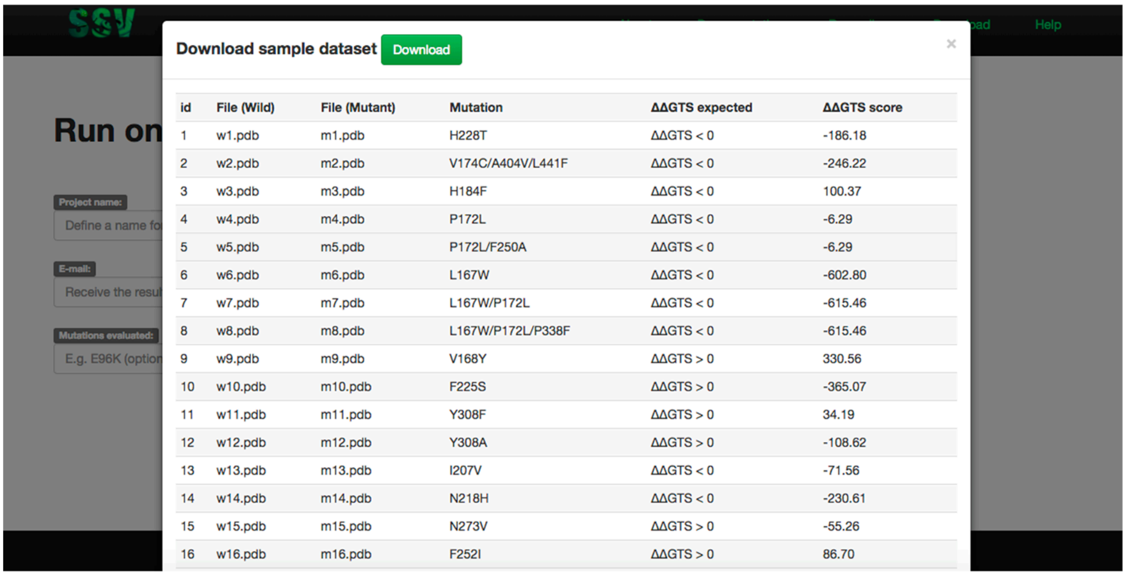

id	File (Wild)	File (Mutant)	Mutation	ΔΔGTS expected	ΔΔGTS score
1	w1.pdb	m1.pdb	H228T	ΔΔGTS < 0	-186.18
2	w2.pdb	m2.pdb	V174C/A404V/L441F	ΔΔGTS < 0	-246.22
3	w3.pdb	m3.pdb	H184F	ΔΔGTS < 0	100.37
4	w4.pdb	m4.pdb	P172L	ΔΔGTS < 0	-6.29
5	w5.pdb	m5.pdb	P172L/F250A	ΔΔGTS < 0	-6.29
6	w6.pdb	m6.pdb	L167W	ΔΔGTS < 0	-602.80
7	w7.pdb	m7.pdb	L167W/P172L	ΔΔGTS < 0	-615.46
8	w8.pdb	m8.pdb	L167W/P172L/P338F	ΔΔGTS < 0	-615.46
9	w9.pdb	m9.pdb	V168Y	ΔΔGTS > 0	330.56
10	w10.pdb	m10.pdb	F225S	ΔΔGTS > 0	-365.07
11	w11.pdb	m11.pdb	Y308F	ΔΔGTS > 0	34.19
12	w12.pdb	m12.pdb	Y308A	ΔΔGTS > 0	-108.62
13	w13.pdb	m13.pdb	I207V	ΔΔGTS < 0	-71.56
14	w14.pdb	m14.pdb	N218H	ΔΔGTS < 0	-230.61
15	w15.pdb	m15.pdb	N273V	ΔΔGTS > 0	-55.26
16	w16.pdb	m16.pdb	F252I	ΔΔGTS > 0	86.70

Running an example (H228T)

Now, we will show how to run the first example of the sample database. The mutation H228T, where detected for a non-tolerant β-glucosidase and improved its glucose tolerance. For this reason, we expected a ΔΔSSV negative.

Download the dataset:

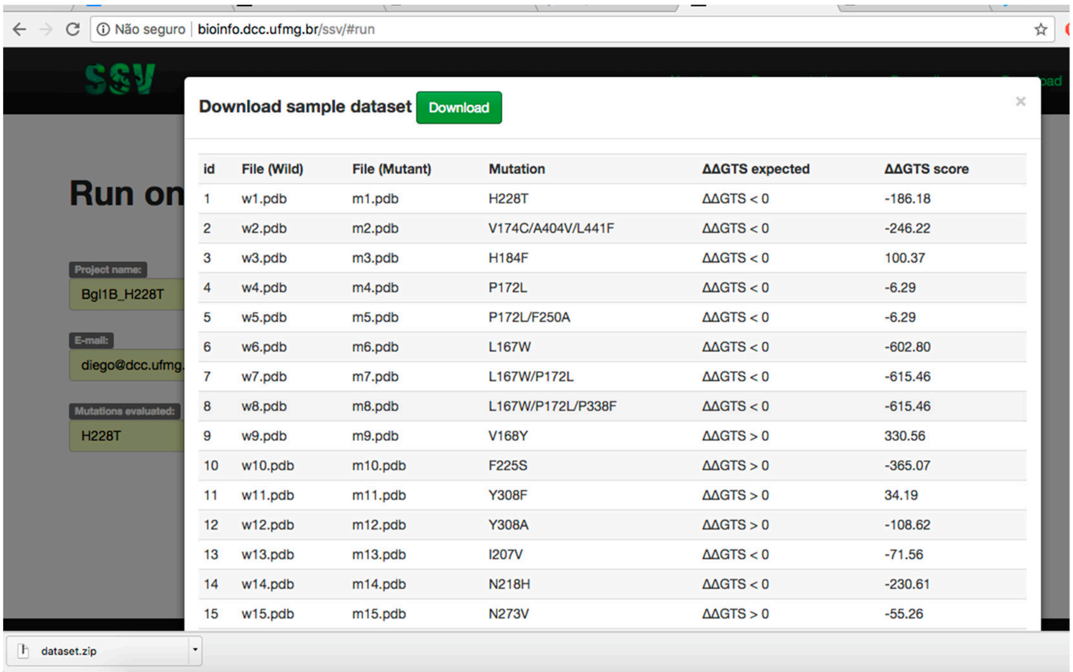

id	File (Wild)	File (Mutant)	Mutation	ΔΔGTS expected	ΔΔGTS score
1	w1.pdb	m1.pdb	H228T	ΔΔGTS < 0	-186.18
2	w2.pdb	m2.pdb	V174C/A404V/L441F	ΔΔGTS < 0	-246.22
3	w3.pdb	m3.pdb	H184F	ΔΔGTS < 0	100.37
4	w4.pdb	m4.pdb	P172L	ΔΔGTS < 0	-6.29
5	w5.pdb	m5.pdb	P172L/F250A	ΔΔGTS < 0	-6.29
6	w6.pdb	m6.pdb	L167W	ΔΔGTS < 0	-602.80
7	w7.pdb	m7.pdb	L167W/P172L	ΔΔGTS < 0	-615.46
8	w8.pdb	m8.pdb	L167W/P172L/P338F	ΔΔGTS < 0	-615.46
9	w9.pdb	m9.pdb	V168Y	ΔΔGTS > 0	330.56
10	w10.pdb	m10.pdb	F225S	ΔΔGTS > 0	-365.07
11	w11.pdb	m11.pdb	Y308F	ΔΔGTS > 0	34.19
12	w12.pdb	m12.pdb	Y308A	ΔΔGTS > 0	-108.62
13	w13.pdb	m13.pdb	I207V	ΔΔGTS < 0	-71.56
14	w14.pdb	m14.pdb	N218H	ΔΔGTS < 0	-230.61
15	w15.pdb	m15.pdb	N273V	ΔΔGTS > 0	-55.26

Extract the files:

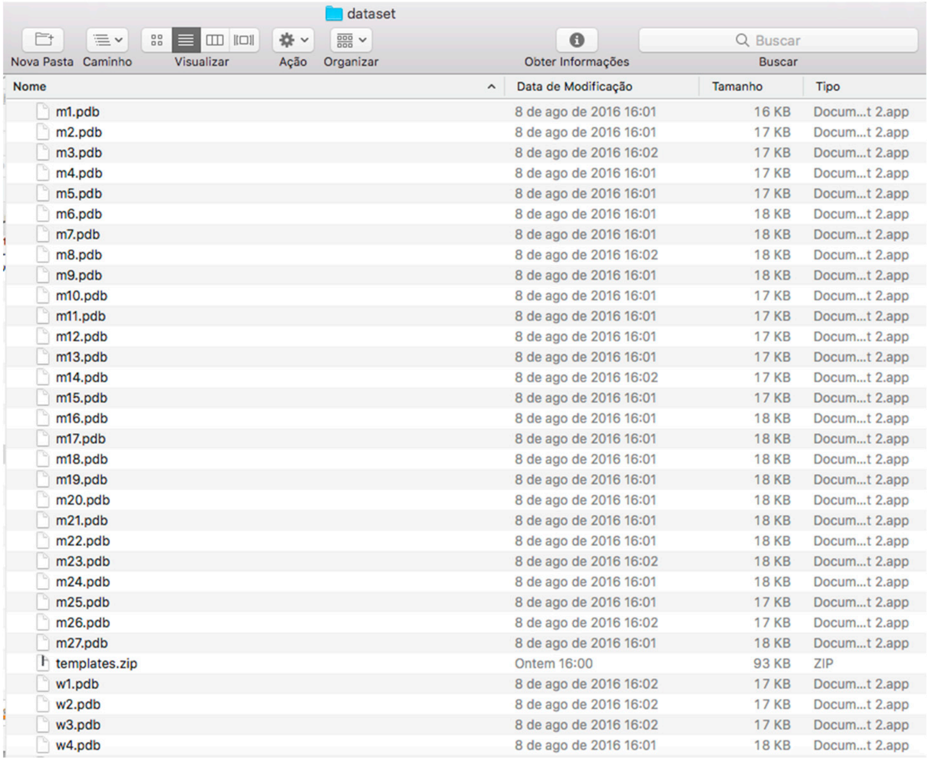

Nome	Data de Modificação	Tamanho	Tipo
m1.pdb	8 de ago de 2016 16:01	16 KB	Docum...t 2.app
m2.pdb	8 de ago de 2016 16:01	17 KB	Docum...t 2.app
m3.pdb	8 de ago de 2016 16:02	17 KB	Docum...t 2.app
m4.pdb	8 de ago de 2016 16:01	17 KB	Docum...t 2.app
m5.pdb	8 de ago de 2016 16:01	17 KB	Docum...t 2.app
m6.pdb	8 de ago de 2016 16:01	18 KB	Docum...t 2.app
m7.pdb	8 de ago de 2016 16:01	18 KB	Docum...t 2.app
m8.pdb	8 de ago de 2016 16:02	18 KB	Docum...t 2.app
m9.pdb	8 de ago de 2016 16:01	18 KB	Docum...t 2.app
m10.pdb	8 de ago de 2016 16:01	17 KB	Docum...t 2.app
m11.pdb	8 de ago de 2016 16:01	17 KB	Docum...t 2.app
m12.pdb	8 de ago de 2016 16:01	17 KB	Docum...t 2.app
m13.pdb	8 de ago de 2016 16:01	17 KB	Docum...t 2.app
m14.pdb	8 de ago de 2016 16:02	17 KB	Docum...t 2.app
m15.pdb	8 de ago de 2016 16:01	17 KB	Docum...t 2.app
m16.pdb	8 de ago de 2016 16:01	18 KB	Docum...t 2.app
m17.pdb	8 de ago de 2016 16:01	18 KB	Docum...t 2.app
m18.pdb	8 de ago de 2016 16:01	18 KB	Docum...t 2.app
m19.pdb	8 de ago de 2016 16:01	18 KB	Docum...t 2.app
m20.pdb	8 de ago de 2016 16:01	18 KB	Docum...t 2.app
m21.pdb	8 de ago de 2016 16:01	18 KB	Docum...t 2.app
m22.pdb	8 de ago de 2016 16:01	18 KB	Docum...t 2.app
m23.pdb	8 de ago de 2016 16:02	18 KB	Docum...t 2.app
m24.pdb	8 de ago de 2016 16:01	18 KB	Docum...t 2.app
m25.pdb	8 de ago de 2016 16:01	17 KB	Docum...t 2.app
m26.pdb	8 de ago de 2016 16:02	17 KB	Docum...t 2.app
m27.pdb	8 de ago de 2016 16:01	18 KB	Docum...t 2.app
templates.zip	Ontem 16:00	93 KB	ZIP
w1.pdb	8 de ago de 2016 16:02	17 KB	Docum...t 2.app
w2.pdb	8 de ago de 2016 16:02	17 KB	Docum...t 2.app
w3.pdb	8 de ago de 2016 16:02	17 KB	Docum...t 2.app
w4.pdb	8 de ago de 2016 16:01	18 KB	Docum...t 2.app

Run SSV using w1.pdb (wild), m1.pdb (mutant), and templates.zip (23 templates):

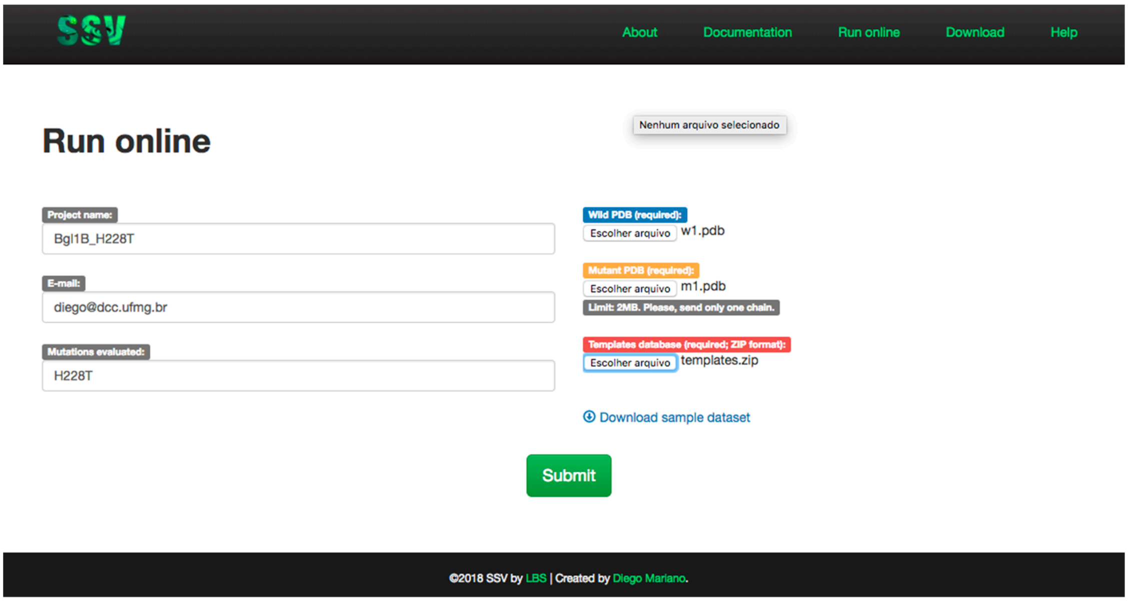

After submitting the data, SSV online will process your requisition. A unique ID will be created for your project. When the process finishes, you can click on the link.

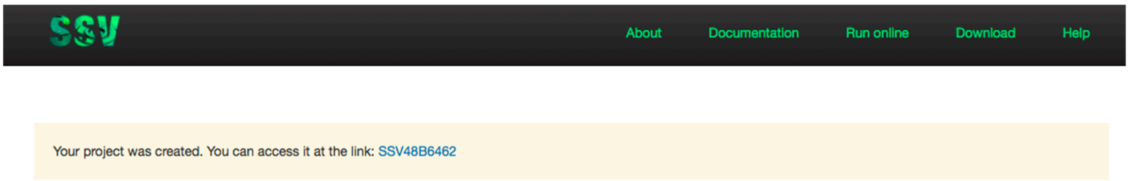

While SSV runs your project, you will receive this message:

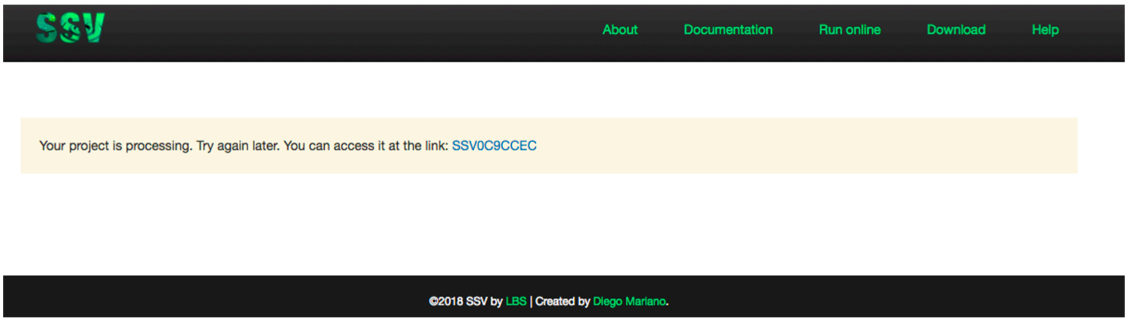

In the end, you will be redirected to the individual page of the project. This page is identified by the unique ID, described under the project name (in the green section).
Below the green section, there are three important sectors:

1. The results of the ΔΔSSV calculation;
2. Wild visualization;
3. Mutant visualization.

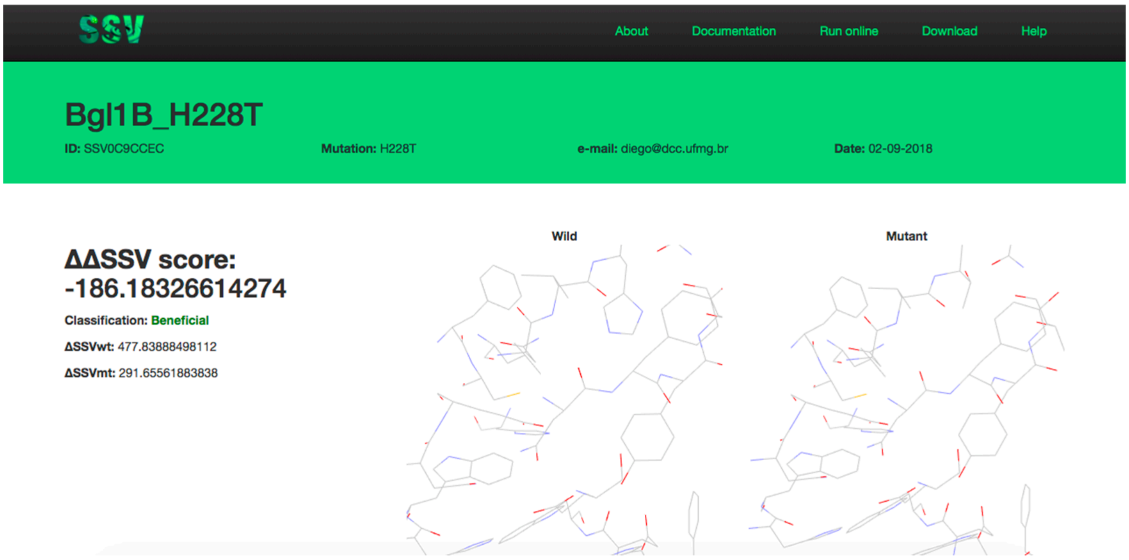

In the main panel, it is shown:

- ΔΔSSV score;
- Classification;
- ΔSSV_{Wt};
- ΔSSV_{Mt}.

Table S1. Origin of β-glucosidases from Betagdb used as templates in SSV method.

#	Organism	UniProt ID	PDB ID	Family	K_i	IC50	Source
1	*Bacillus subtilis*	I3QIG4	ND*	GH1	1,900	ND*	(Chamoli *et al.*, 2016)
2	*Uncultured bacterium* (Metagenome China South Sea)	D5KX75	ND*	GH1	ND*	1,000	(Y. Yang *et al.*, 2015)
3	*Uncultured bacterium* (Metagenome Turpan Depression)	A0A0F7KKB7	ND*	GH1	ND*	3,500	(Cao *et al.*, 2015)
4	*Exiguobacterium antarcticum* B7	K0A8J9	5DT5	GH1	ND*	1,000	(Crespim *et al.*, 2016)
5	*Uncultured bacterium* (Metagenome Kusaya gravy)	HV348683.1	ND*	GH1	ND*	> 750	(Uchiyama *et al.*, 2015)
6	*Thermoanaerobacterium aotearoense*	A0A0H4NXH8	ND*	GH1	800	ND*	(F. Yang *et al.*, 2015)
7	*Talaromyces funiculosus (Penicillium funiculosum)*	K4KB38	ND*	GH3	ND*	400	(Ramani *et al.*, 2015)
8	*Humicola grisea* var. *thermoidea*	O93784	4MDO	GH1	ND*	> 450	(de Giuseppe *et al.*, 2014); (Benoliel *et al.*, 2010)
9	*Uncultured bacterium* (Metagenome soil)	K4I4U1	ND*	GH1	ND*	> 300	(Lu *et al.*, 2013)
10	*Thermoanaerobacterium thermosaccharolyticum*	D9TR57	ND*	GH1	600	ND*	(Pei *et al.*, 2012)
11	*Fervidobacterium islandicum*	G8YZD7	ND*	GH1	211	ND*	(Jabbour *et al.*, 2012)
12	*Mucor circinelloides*	ND*	ND*	GH3	ND*	> 140	(Huang *et al.*, 2014)
13	*Hypocrea jecorina (Trichoderma reesei)*	O93785	ND*	GH1	ND*	650	(Guo *et al.*, 2016)
14	*Thermotoga naphthophila*	D2C6W2	ND*	GH1	1200	ND*	(Akram *et al.*, 2016)
15	*Caldicellulosiruptor bescii*	B9MNR1	ND*	GH1	113.8	ND*	(Bai *et al.*, 2013)

16	*Neurospora crassa*	U9W8B8	ND*	GH1	ND*	950	(Meleiro *et al.*, 2015)
17	*Pyrococcus furiosus*	E7FHY4	3APG	GH1	207	ND*	(Cota *et al.*, 2015)
18	*Thermotoga petrophila*	A5IL97	ND*	GH1	1100	ND*	(Cota *et al.*, 2015)
19	*Acidilobus saccharovorans*	D9PZ08	4HA3	GH1	500	ND*	(Gumerov *et al.*, 2015)
20	*Uncultured bacterium* (Metagenome hydrothermal spring)	W8W3B8	ND*	GH1	150	ND*	(Schröder *et al.*, 2014)
21	*Neotermes koshunensis*	Q8T0W7	3AHZ	GH1	ND*	> 1000	(Uchima *et al.*, 2011); (de Giuseppe *et al.*, 2014)
22	*Thermoanaerobacter brockii*	Q60026	ND*	GH1	200	ND*	(Breves *et al.*, 1997)
23	*Nasutitermes takasagoensis*	D0VYR9	ND*	GH1	ND*	> 600	(Uchima *et al.*, 2012)

Source: http://bioinfo.dcc.ufmg.br/betagdb

*ND: not determined.

Table S2. ΔΔSSV values for Bgl1B's mutations.

Mutation represents the residue inserted. Template for the wild type was *Thermoanaerobacter brockii.* Mutations classified by SSV presented ΔΔSSV lower than zero. We used Clustal Omega to detect residues 100% conserved. They are H125, N169, E170, Y298, E353, and W399. We removed 9 mutations (Y298: 6; E353: 1; and W399: 2). We evaluated residues allowed for the position using SIFT. Only 19 mutations were classified. Then we removed four mutations considered highly destabilizing by mCSM. A total of 15 mutations were proposed for Bgl1B.

**Mutation**	**ΔΔSSV**	**Mutant's template**	**Mutations classified by**			
			**SSV**	**CLUSTAL**	**SIFT**	**mCSM**
ALA228	-270.08	*Caldicellulosiruptor bescii*	x	x	x	x
CYS228	-219.89	*Caldicellulosiruptor bescii*	x	x	x	x
THR228	-186.18	*Caldicellulosiruptor bescii*	x	x	x	x
GLY228	-185.37	*Caldicellulosiruptor bescii*	x	x	x	
ALA297	-166.47	*Caldicellulosiruptor bescii*	x	x		
THR172	-158.78	*Thermoanaerobacterium thermosaccharolyticum*	x	x		
SER228	-150.62	*Caldicellulosiruptor bescii*	x	x	x	
CYS297	-149.21	*Caldicellulosiruptor bescii*	x	x		
THR297	-146.10	*Caldicellulosiruptor bescii*	x	x		

ASN172	-133.18	*Thermoanaerobacterium thermosaccharolyticum*	x	x		
ASN297	-129.86	*Thermoanaerobacterium thermosaccharolyticum*	x	x		
SER297	-124.83	*Caldicellulosiruptor bescii*	x	x		
GLN172	-122.81	*Thermoanaerobacterium thermosaccharolyticum*	x	x		
CYS172	-122.79	*Thermoanaerobacterium thermosaccharolyticum*	x	x		
VAL228	-121.53	*Caldicellulosiruptor bescii*	x	x	x	x
LEU415	-119.77	*Fervidobacterium islandicum*	x	x		
GLY297	-118.16	*Caldicellulosiruptor bescii*	x	x		
ILE415	-112.61	*Fervidobacterium islandicum*	x	x		
GLY172	-110.12	*Thermoanaerobacterium aotearoense*	x	x		
SER172	-110.05	*Thermoanaerobacterium thermosaccharolyticum*	x	x		
ALA172	-100.41	*Thermoanaerobacterium aotearoense*	x	x		
HIS298	-100.34	*Thermoanaerobacter brockii*	x			
PHE399	-92.67	*Metagenome China South Sea*	x			
LEU184	-80.10	*Fervidobacterium islandicum*	x	x		
HIS297	-79.86	*Thermoanaerobacter brockii*	x	x		
PRO228	-76.56	*Caldicellulosiruptor bescii*	x	x	x	x
LYS172	-75.20	*Thermoanaerobacterium thermosaccharolyticum*	x	x	x	x
VAL415	-74.93	*Fervidobacterium islandicum*	x	x		
ILE184	-74.34	*Fervidobacterium islandicum*	x	x		
GLN297	-68.63	*Thermoanaerobacterium thermosaccharolyticum*	x	x		
HIS415	-67.62	*Thermoanaerobacter brockii*	x	x		
MET415	-67.20	*Fervidobacterium islandicum*	x	x		
PRO297	-62.03	*Caldicellulosiruptor bescii*	x	x		
SER415	-61.96	*Metagenome China South Sea*	x	x		
VAL172	-60.38	*Thermoanaerobacterium thermosaccharolyticum*	x	x	x	x
PRO172	-57.35	*Thermoanaerobacterium thermosaccharolyticum*	x	x		
ASP172	-53.10	*Thermoanaerobacterium thermosaccharolyticum*	x	x		
TYR399	-52.16	*Metagenome China South Sea*	x			
MET172	-51.94	*Thermoanaerobacterium thermosaccharolyticum*	x	x		
CYS415	-51.51	*Metagenome China South Sea*	x	x		
MET297	-51.22	*Caldicellulosiruptor bescii*	x	x		
PRO415	-51.04	*Fervidobacterium islandicum*	x	x		
VAL297	-48.08	*Caldicellulosiruptor bescii*	x	x		
ASP415	-47.73	*Metagenome China South Sea*	x	x		
THR415	-44.78	*Metagenome China South Sea*	x	x		

MET228	-44.19	*Caldicellulosiruptor bescii*	x	x	x	x
GLU172	-42.44	*Thermoanaerobacterium thermosaccharolyticum*	x	x		
GLY415	-40.79	*Metagenome China South Sea*	x	x		
TYR126	-40.40	*Thermoanaerobacterium thermosaccharolyticum*	x	x		
THR298	-40.27	*Caldicellulosiruptor bescii*	x			
GLY173	-39.82	*Thermotoga naphthophila*	x	x		
ALA184	-36.31	*Caldicellulosiruptor bescii*	x	x		
LYS297	-35.97	*Caldicellulosiruptor bescii*	x	x		
ALA415	-32.52	*Metagenome China South Sea*	x	x		
ASP406	-31.51	*Thermoanaerobacter brockii*	x	x		
PHE407	-31.44	*Metagenome China South Sea*	x	x		
GLN228	-30.10	*Caldicellulosiruptor bescii*	x	x	x	x
VAL184	-28.59	*Fervidobacterium islandicum*	x	x		
LYS227	-25.50	*Thermoanaerobacter brockii*	x	x	x	
GLY184	-22.77	*Caldicellulosiruptor bescii*	x	x		
PRO184	-18.33	*Caldicellulosiruptor bescii*	x	x		
SER246	-17.29	*Thermoanaerobacter brockii*	x	x	x	x
SER299	-17.18	*Thermoanaerobacter brockii*	x	x	x	x
ASP353	-16.60	*Thermoanaerobacter brockii*	x			
THR184	-15.82	*Thermoanaerobacterium thermosaccharolyticum*	x	x		
LEU298	-15.68	*Metagenome Turpan Depression*	x			
LYS415	-14.08	*Fervidobacterium islandicum*	x	x		
ASN228	-13.91	*Caldicellulosiruptor bescii*	x	x	x	x
MET184	-13.13	*Fervidobacterium islandicum*	x	x		
ILE298	-13.08	*Metagenome Turpan Depression*	x			
GLN415	-11.25	*Thermoanaerobacterium thermosaccharolyticum*	x	x		
ASN298	-10.71	*Caldicellulosiruptor bescii*	x			
ASP297	-9.86	*Caldicellulosiruptor bescii*	x	x		
ASN415	-9.46	*Metagenome China South Sea*	x	x		
VAL177	-8.65	*Thermoanaerobacter brockii*	x	x		
GLU415	-7.64	*Fervidobacterium islandicum*	x	x		
CYS184	-7.40	*Metagenome China South Sea*	x	x		
PRO177	-6.97	*Thermoanaerobacter brockii*	x	x		
MET227	-5.29	*Thermoanaerobacter brockii*	x	x	x	x
ILE172	-4.40	*Thermoanaerobacterium thermosaccharolyticum*	x	x	x	x
SER298	-3.64	*Caldicellulosiruptor bescii*	x			

THR246	-2.44	*Thermoanaerobacter brockii*	x	x	x	x
GLN227	-1.88	*Thermoanaerobacter brockii*	x	x	x	
CYS299	-1.63	*Thermoanaerobacter brockii*	x	x		
ILE177	-1.21	*Thermoanaerobacter brockii*	x	x		
SER184	-0.70	*Metagenome China South Sea*	x	x		
GLU297	0.30	*Caldicellulosiruptor bescii*				
GLY169	0.52	*Exiguobacterium antarcticum B7*				
PHE297	0.82	*Thermoanaerobacter brockii*				
CYS246	0.91	*Thermoanaerobacter brockii*				
ARG227	1.29	*Thermoanaerobacter brockii*				
THR301	2.29	*Thermoanaerobacter brockii*				
ALA246	2.62	*Thermoanaerobacter brockii*				
SER169	3.50	*Exiguobacterium antarcticum B7*				
ASP170	3.78	*Thermoanaerobacter brockii*				
LEU227	4.12	*Thermoanaerobacter brockii*				
GLY299	4.23	*Thermoanaerobacter brockii*				
LEU172	4.28	*Thermoanaerobacterium thermosaccharolyticum*				
HIS399	4.57	*Metagenome China South Sea*				
ILE125	5.63	*Fervidobacterium islandicum*				
THR169	5.70	*Thermoanaerobacter brockii*				
SER301	7.58	*Thermoanaerobacter brockii*				
LYS184	7.76	*Fervidobacterium islandicum*				
ALA298	10.63	*Caldicellulosiruptor bescii*				
PRO227	11.20	*Thermoanaerobacter brockii*				
ALA299	11.44	*Thermoanaerobacter brockii*				
ALA169	12.69	*Exiguobacterium antarcticum B7*				
CYS298	12.72	*Caldicellulosiruptor bescii*				
HIS172	14.35	*Thermoanaerobacter brockii*				
GLU227	16.14	*Thermoanaerobacter brockii*				
THR296	16.34	*Thermoanaerobacter brockii*				
THR226	17.10	*Thermoanaerobacter brockii*				
PHE298	17.29	*Thermoanaerobacter brockii*				
GLN246	18.74	*Thermoanaerobacter brockii*				
GLU246	19.16	*Thermoanaerobacter brockii*				
ALA173	19.43	*Thermotoga naphthophila*				
PHE126	19.86	*Caldicellulosiruptor bescii*				

HIS126	20.49	*Caldicellulosiruptor bescii*
CYS227	22.67	*Thermoanaerobacter brockii*
MET177	23.11	*Thermoanaerobacter brockii*
ASP228	23.95	*Caldicellulosiruptor bescii*
ARG297	25.81	*Thermoanaerobacterium thermosaccharolyticum*
MET125	26.91	*Fervidobacterium islandicum*
ASP184	27.94	*Metagenome China South Sea*
ASP246	29.15	*Thermoanaerobacter brockii*
LEU297	29.25	*Caldicellulosiruptor bescii*
LYS406	29.66	*Thermoanaerobacter brockii*
ASN246	29.69	*Thermoanaerobacter brockii*
ILE227	30.17	*Thermoanaerobacter brockii*
GLN301	30.77	*Thermoanaerobacter brockii*
LEU228	32.34	*Metagenome Turpan Depression*
PRO246	32.82	*Thermoanaerobacter brockii*
ALA406	35.02	*Thermoanaerobacter brockii*
CYS301	35.20	*Thermoanaerobacter brockii*
ALA227	35.51	*Thermoanaerobacter brockii*
TYR415	36.43	*Thermoanaerobacter brockii*
TYR407	37.26	*Metagenome China South Sea*
LYS228	37.89	*Caldicellulosiruptor bescii*
ASP301	37.96	*Thermoanaerobacter brockii*
ILE228	38.82	*Caldicellulosiruptor bescii*
LEU125	40.27	*Fervidobacterium islandicum*
ALA125	41.28	*Caldicellulosiruptor bescii*
ILE297	41.34	*Caldicellulosiruptor bescii*
GLY406	42.61	*Thermotoga naphthophila*
VAL246	42.65	*Thermoanaerobacter brockii*
GLY125	42.75	*Caldicellulosiruptor bescii*
LYS301	43.87	*Thermoanaerobacter brockii*
CYS406	44.25	*Thermoanaerobacter brockii*
PRO298	45.80	*Metagenome Turpan Depression*
ASN184	46.47	*Thermoanaerobacterium thermosaccharolyticum*
SER296	48.70	*Thermoanaerobacter brockii*
GLN184	49.02	*Thermoanaerobacterium thermosaccharolyticum*
LYS125	51.09	*Fervidobacterium islandicum*

ALA301	51.14	*Thermoanaerobacter brockii*
GLY301	52.23	*Thermoanaerobacter brockii*
ASN299	54.45	*Thermoanaerobacter brockii*
GLU301	55.26	*Thermoanaerobacter brockii*
SER226	57.79	*Thermoanaerobacter brockii*
CYS169	58.57	*Exiguobacterium antarcticum B7*
GLN298	59.65	*Caldicellulosiruptor bescii*
PRO125	63.05	*Caldicellulosiruptor bescii*
GLY298	63.90	*Caldicellulosiruptor bescii*
VAL298	64.25	*Metagenome Turpan Depression*
VAL125	66.21	*Fervidobacterium islandicum*
CYS125	66.58	*Caldicellulosiruptor bescii*
GLN299	68.93	*Thermoanaerobacter brockii*
ARG172	69.21	*Thermoanaerobacterium thermosaccharolyticum*
ASN227	69.92	*Thermoanaerobacter brockii*
HIS407	70.31	*Fervidobacterium islandicum*
LYS177	73.09	*Thermoanaerobacter brockii*
THR227	74.83	*Thermoanaerobacter brockii*
GLY227	76.31	*Metagenome China South Sea*
VAL301	76.58	*Thermoanaerobacter brockii*
ASP227	77.35	*Thermoanaerobacter brockii*
ALA177	77.54	*Metagenome China South Sea*
MET301	77.60	*Thermoanaerobacter brockii*
ALA296	77.73	*Exiguobacterium antarcticum B7*
ARG301	79.47	*Thermoanaerobacter brockii*
ALA226	79.56	*Exiguobacterium antarcticum B7*
TYR172	81.63	*Thermoanaerobacter brockii*
MET246	82.13	*Thermoanaerobacter brockii*
ALA353	87.69	*Thermotoga naphthophila*
ASP299	88.94	*Thermoanaerobacter brockii*
GLY296	88.99	*Exiguobacterium antarcticum B7*
ILE246	91.59	*Thermoanaerobacter brockii*
CYS296	91.60	*Exiguobacterium antarcticum B7*
GLY226	93.23	*Thermotoga naphthophila*
GLY353	95.51	*Thermotoga naphthophila*
ARG415	97.16	*Thermoanaerobacterium thermosaccharolyticum*

GLU184	98.79	*Fervidobacterium islandicum*
ILE301	98.85	*Thermoanaerobacter brockii*
SER173	99.17	*Thermoanaerobacter brockii*
CYS226	99.46	*Exiguobacterium antarcticum B7*
SER406	99.63	*Thermoanaerobacter brockii*
PHE184	100.37	*Thermoanaerobacter brockii*
PRO301	100.79	*Thermoanaerobacter brockii*
THR173	101.09	*Thermoanaerobacter brockii*
THR125	102.72	*Metagenome China South Sea*
SER125	103.12	*Metagenome China South Sea*
LEU301	103.32	*Thermoanaerobacter brockii*
CYS177	103.64	*Metagenome China South Sea*
LEU246	103.96	*Thermoanaerobacter brockii*
GLU299	104.40	*Thermoanaerobacter brockii*
SER227	105.56	*Metagenome China South Sea*
MET298	106.41	*Metagenome Turpan Depression*
LYS246	106.63	*Thermoanaerobacter brockii*
GLN125	107.01	*Fervidobacterium islandicum*
GLU125	107.06	*Fervidobacterium islandicum*
PHE125	112.75	*Thermoanaerobacter brockii*
MET299	113.06	*Thermoanaerobacter brockii*
GLN406	114.60	*Thermoanaerobacter brockii*
ASN125	115.07	*Metagenome China South Sea*
VAL299	116.10	*Fervidobacterium islandicum*
VAL406	116.20	*Thermoanaerobacter brockii*
PRO299	116.82	*Fervidobacterium islandicum*
GLU228	118.99	*Caldicellulosiruptor bescii*
ILE299	119.86	*Fervidobacterium islandicum*
LEU299	121.06	*Fervidobacterium islandicum*
GLN353	121.19	*Thermoanaerobacter brockii*
LYS298	123.40	*Caldicellulosiruptor bescii*
PRO169	123.47	*Exiguobacterium antarcticum B7*
TYR184	124.56	*Thermoanaerobacter brockii*
GLN296	125.75	*Thermoanaerobacter brockii*
PRO406	128.29	*Thermoanaerobacter brockii*
ASP125	130.71	*Metagenome China South Sea*

THR406	133.01	*Thermoanaerobacter brockii*
ARG228	135.05	*Neotermes koshunensis*
THR177	135.39	*Metagenome China South Sea*
ASP298	135.96	*Caldicellulosiruptor bescii*
LYS299	142.59	*Thermoanaerobacter brockii*
PRO173	143.95	*Fervidobacterium islandicum*
GLY177	149.61	*Metagenome China South Sea*
VAL173	154.00	*Fervidobacterium islandicum*
GLN177	160.01	*Metagenome China South Sea*
CYS353	161.61	*Thermoanaerobacter brockii*
LYS296	165.52	*Fervidobacterium islandicum*
GLU177	169.66	*Thermoanaerobacter brockii*
ALA170	170.13	*Thermotoga naphthophila*
LYS226	170.17	*Fervidobacterium islandicum*
MET406	171.05	*Thermoanaerobacter brockii*
TYR125	172.72	*Thermoanaerobacter brockii*
GLN169	177.88	*Thermoanaerobacter brockii*
ASP177	179.85	*Metagenome China South Sea*
VAL169	183.96	*Fervidobacterium islandicum*
GLN170	184.48	*Thermoanaerobacter brockii*
ARG246	185.45	*Thermoanaerobacter brockii*
ASN177	187.18	*Metagenome China South Sea*
GLU298	192.83	*Metagenome Turpan Depression*
SER177	195.24	*Metagenome China South Sea*
PRO296	195.63	*Fervidobacterium islandicum*
LYS169	196.08	*Fervidobacterium islandicum*
PRO226	198.56	*Fervidobacterium islandicum*
VAL296	199.05	*Fervidobacterium islandicum*
VAL226	203.28	*Fervidobacterium islandicum*
GLN226	207.02	*Thermoanaerobacter brockii*
PHE228	207.89	*Thermoanaerobacter brockii*
ASN406	207.97	*Thermoanaerobacter brockii*
THR353	208.61	*Thermoanaerobacter brockii*
SER353	212.35	*Thermotoga naphthophila*
TRP125	213.51	*Trichoderma reesei*
ARG299	213.89	*Thermoanaerobacter brockii*

LEU406	214.16	*Fervidobacterium islandicum*
ARG184	214.40	*Thermoanaerobacterium thermosaccharolyticum*
ARG296	219.42	*Fervidobacterium islandicum*
MET173	221.35	*Fervidobacterium islandicum*
GLY170	224.39	*Thermotoga naphthophila*
TRP298	226.34	*Neurospora crassa*
ILE169	228.03	*Fervidobacterium islandicum*
MET296	229.39	*Fervidobacterium islandicum*
TRP184	229.41	*Trichoderma reesei*
TRP415	231.09	*Trichoderma reesei*
ILE406	234.39	*Fervidobacterium islandicum*
CYS170	234.73	*Thermotoga naphthophila*
MET169	235.71	*Fervidobacterium islandicum*
HIS246	238.43	*Thermoanaerobacter brockii*
VAL353	241.57	*Thermotoga naphthophila*
PRO353	243.48	*Thermotoga naphthophila*
LEU169	245.93	*Fervidobacterium islandicum*
LEU296	247.88	*Fervidobacterium islandicum*
ASP169	248.04	*Exiguobacterium antarcticum B7*
ASP296	249.37	*Exiguobacterium antarcticum B7*
ASP173	251.62	*Thermoanaerobacter brockii*
MET226	252.15	*Fervidobacterium islandicum*
PHE177	252.71	*Trichoderma reesei*
ILE173	261.97	*Fervidobacterium islandicum*
TYR177	268.32	*Trichoderma reesei*
GLY126	269.84	*Metagenome hydrothermal spring*
PRO170	270.55	*Thermotoga naphthophila*
TRP246	272.41	*Trichoderma reesei*
ASP226	272.45	*Exiguobacterium antarcticum B7*
ILE296	273.62	*Fervidobacterium islandicum*
MET353	277.15	*Thermotoga naphthophila*
ALA126	279.33	*Metagenome hydrothermal spring*
HIS177	280.17	*Trichoderma reesei*
LEU173	280.89	*Fervidobacterium islandicum*
ILE226	282.35	*Fervidobacterium islandicum*
TYR228	283.30	*Thermoanaerobacter brockii*

MET126	286.09	*Metagenome soil*
ASN173	286.89	*Thermoanaerobacter brockii*
LEU226	289.21	*Fervidobacterium islandicum*
LEU353	291.07	*Fervidobacterium islandicum*
HIS299	291.91	*Neurospora crassa*
SER126	292.73	*Metagenome hydrothermal spring*
TRP299	293.73	*Neurospora crassa*
ILE353	295.19	*Fervidobacterium islandicum*
ASN353	295.30	*Thermoanaerobacter brockii*
THR170	295.46	*Thermotoga naphthophila*
TRP227	301.08	*Trichoderma reesei*
GLN173	302.45	*Thermoanaerobacter brockii*
TRP177	303.93	*Trichoderma reesei*
PRO126	304.61	*Metagenome soil*
TYR299	307.00	*Neurospora crassa*
VAL170	308.02	*Thermotoga naphthophila*
ALA399	308.98	*Caldicellulosiruptor bescii*
PHE299	309.34	*Trichoderma reesei*
TRP297	309.59	*Thermoanaerobacter brockii*
VAL126	309.62	*Metagenome soil*
ILE126	313.06	*Metagenome soil*
THR126	313.55	*Metagenome hydrothermal spring*
CYS126	315.42	*Metagenome hydrothermal spring*
GLU296	315.79	*Thermoanaerobacter brockii*
LEU399	316.31	*Metagenome Turpan Depression*
TRP172	316.52	*Neurospora crassa*
SER170	317.40	*Thermotoga naphthophila*
ILE399	319.56	*Metagenome Turpan Depression*
PRO399	321.99	*Caldicellulosiruptor bescii*
CYS399	322.50	*Caldicellulosiruptor bescii*
ARG226	323.19	*Fervidobacterium islandicum*
LYS173	323.41	*Fervidobacterium islandicum*
LEU126	323.88	*Metagenome soil*
TRP301	325.10	*Trichoderma reesei*
GLY399	325.33	*Caldicellulosiruptor bescii*
THR399	326.94	*Caldicellulosiruptor bescii*

TRP228	328.97	*Trichoderma reesei*
VAL399	336.64	*Caldicellulosiruptor bescii*
SER399	340.59	*Caldicellulosiruptor bescii*
ASN399	342.40	*Caldicellulosiruptor bescii*
PHE246	346.48	*Neurospora crassa*
ILE170	346.51	*Fervidobacterium islandicum*
ARG177	346.99	*Thermoanaerobacter brockii*
ASP399	349.59	*Caldicellulosiruptor bescii*
LYS353	351.24	*Thermoanaerobacter brockii*
TYR246	351.72	*Trichoderma reesei*
PHE301	352.19	*Neurospora crassa*
ARG298	353.80	*Caldicellulosiruptor bescii*
MET399	356.19	*Caldicellulosiruptor bescii*
ASN126	358.32	*Metagenome hydrothermal spring*
GLU173	359.91	*Thermoanaerobacter brockii*
HIS173	361.75	*Trichoderma reesei*
GLU226	362.39	*Fervidobacterium islandicum*
TYR301	363.13	*Neurospora crassa*
HIS301	363.61	*Neurospora crassa*
PHE227	365.26	*Trichoderma reesei*
GLU169	366.43	*Thermoanaerobacter brockii*
GLN399	368.18	*Caldicellulosiruptor bescii*
TYR227	368.98	*Trichoderma reesei*
ARG125	369.09	*Fervidobacterium islandicum*
GLU399	373.98	*Caldicellulosiruptor bescii*
LEU170	383.52	*Fervidobacterium islandicum*
MET170	387.60	*Thermotoga naphthophila*
ASP126	390.42	*Metagenome hydrothermal spring*
HIS227	391.97	*Neurospora crassa*
PHE173	392.31	*Trichoderma reesei*
ARG126	396.76	*Metagenome hydrothermal spring*
LYS399	403.12	*Caldicellulosiruptor bescii*
ASN170	431.86	*Metagenome China South Sea*
GLU126	432.50	*Metagenome Kusaya gravy*
GLN126	433.21	*Metagenome hydrothermal spring*
LYS126	437.83	*Metagenome soil*

PHE406	456.69	*Neurospora crassa*
HIS169	459.45	*Humicola grisea var thermoidea*
LYS170	469.44	*Thermoanaerobacter brockii*
ARG169	475.45	*Fervidobacterium islandicum*
HIS406	475.93	*Neurospora crassa*
TYR406	490.88	*Neurospora crassa*
TYR169	543.94	*Trichoderma reesei*
ILE407	549.17	*Metagenome Turpan Depression*
LEU407	551.85	*Metagenome Turpan Depression*
HIS226	554.71	*Humicola grisea var thermoidea*
PHE169	555.82	*Humicola grisea var thermoidea*
ARG399	573.57	*Caldicellulosiruptor bescii*
ALA407	575.78	*Caldicellulosiruptor bescii*
GLY407	588.38	*Caldicellulosiruptor bescii*
VAL407	588.81	*Metagenome Turpan Depression*
CYS407	589.47	*Caldicellulosiruptor bescii*
PRO407	595.08	*Caldicellulosiruptor bescii*
ARG170	600.83	*Thermoanaerobacterium thermosaccharolyticum*
MET407	600.97	*Metagenome Turpan Depression*
HIS296	610.08	*Trichoderma reesei*
THR407	612.95	*Caldicellulosiruptor bescii*
TYR296	613.25	*Trichoderma reesei*
TYR173	614.06	*Trichoderma reesei*
SER407	617.69	*Caldicellulosiruptor bescii*
ASP407	620.36	*Caldicellulosiruptor bescii*
GLU407	637.99	*Metagenome Turpan Depression*
PHE226	641.36	*Humicola grisea var thermoidea*
LYS407	657.60	*Caldicellulosiruptor bescii*
GLN407	662.02	*Caldicellulosiruptor bescii*
HIS353	664.86	*Trichoderma reesei*
TYR226	666.21	*Trichoderma reesei*
ASN407	671.47	*Caldicellulosiruptor bescii*
ARG406	673.57	*Thermoanaerobacterium thermosaccharolyticum*
PHE296	681.61	*Trichoderma reesei*
PHE353	698.31	*Trichoderma reesei*
ARG173	717.22	*Fervidobacterium islandicum*

ARG353	717.90	*Thermoanaerobacterium thermosaccharolyticum*
TYR353	820.30	*Trichoderma reesei*
ARG407	872.82	*Fervidobacterium islandicum*
HIS170	894.57	*Trichoderma reesei*
PHE170	1,023.87	*Trichoderma reesei*
TRP173	1,052.78	*Trichoderma reesei*
TYR170	1,112.36	*Trichoderma reesei*
TRP169	1,129.74	*Humicola grisea var thermoidea*
TRP226	1,148.79	*Trichoderma reesei*
TRP406	1,221.38	*Trichoderma reesei*
TRP353	1,244.88	*Trichoderma reesei*
TRP296	1,272.51	*Trichoderma reesei*
TRP170	1,739.57	*Trichoderma reesei*

Table S3. Mutations allowed for Bgl1B according to SIFT results.

Residues H125, N169, E170, Y298, E353, and W399 were not evaluated.

Residue	Mutations allowed
**W126**	W
**F172**	i v l Y F W
**C173**	C
**L177**	L
**H184**	H
**N226**	t a r g e s k d Q N
**V227**	y r q c f p s k e m T L A I V
**H228**	w C m p i q d g r H e k n v f l a y S T
**G246**	m c f h y i l n p V r q d t k e G S A
**N296**	N
**Y297**	Y
**T299**	a S T
**N301**	m c f y i h l v p r q k t e g d s A N
**E406**	E
**W407**	W
**F415**	F

Table S4. mCSM result for Bgl1B.

#	PDB File	Chain	Wild Residue	Residue Position	Mutant Residue	RSA (%)	Predicted ΔΔG	Outcome
1	bgl1B.pdb	A	H	228	A	19.1	-1.918	Destabilizing
2	bgl1B.pdb	A	H	228	C	19.1	-0.932	Destabilizing
3	bgl1B.pdb	A	H	228	T	19.1	-1.728	Destabilizing
4	bgl1B.pdb	A	H	228	G	19.1	-2.307	Highly Destabilizing
5	bgl1B.pdb	A	H	228	S	19.1	-2.028	Highly Destabilizing
6	bgl1B.pdb	A	H	228	V	19.1	-1.13	Destabilizing
7	bgl1B.pdb	A	H	228	P	19.1	-1.13	Destabilizing
8	bgl1B.pdb	A	F	172	K	11.6	-1.612	Destabilizing
9	bgl1B.pdb	A	F	172	V	11.6	-1.077	Destabilizing
10	bgl1B.pdb	A	H	228	M	19.1	-0.841	Destabilizing
11	bgl1B.pdb	A	H	228	Q	19.1	-1.568	Destabilizing
12	bgl1B.pdb	A	V	227	K	8.7	-2.049	Highly Destabilizing
13	bgl1B.pdb	A	G	246	S	4.9	-1.662	Destabilizing
14	bgl1B.pdb	A	T	299	S	4.3	-1.707	Destabilizing
15	bgl1B.pdb	A	H	228	N	19.1	-1.951	Destabilizing
16	bgl1B.pdb	A	V	227	M	8.7	-0.782	Destabilizing
17	bgl1B.pdb	A	F	172	I	11.6	-0.619	Destabilizing
18	bgl1B.pdb	A	G	246	T	4.9	-1.433	Destabilizing
19	bgl1B.pdb	A	V	227	Q	8.7	-2.329	Highly Destabilizing

Table S5. Case study 3.

Mutant	Mutation	IF	Classification expected	ΔΔSSV	Link
M1	G39A/W104F/L278A	6.3	Beneficial	-841	http://bioinfo.dcc.ufmg.br/ssv/project/id/SSVC69F173
M2	G39A/T103G/L278A	3.8	Beneficial	-121	http://bioinfo.dcc.ufmg.br/ssv/project/id/SSVCF1AB06
M3	G39A/T103G/W104F/L278A	11.2	Beneficial	-841	http://bioinfo.dcc.ufmg.br/ssv/project/id/SSV84D8A0C
M4	G39A	2.8	Beneficial	150	http://bioinfo.dcc.ufmg.br/ssv/project/id/SSV273B233
M5	G39A/L278A	3.3	Beneficial	-121	http://bioinfo.dcc.ufmg.br/ssv/project/id/SSV2911964
M6	I189A	0.4	Not beneficial	-94	http://bioinfo.dcc.ufmg.br/ssv/project/id/SSVE6997DF
M7	T40A	0.4	Not beneficial	40	http://bioinfo.dcc.ufmg.br/ssv/project/id/SSV6D921F4
M8*	T103G	1.1	Neutral/Beneficial	0	http://bioinfo.dcc.ufmg.br/ssv/project/id/SSV703D402

Figure S1. Residues collected from 1TCA for the case study 3.

Residues at 6.5 Å from the ligand docked at 1TCA (blue); in yellow the catalytic serine (S105); in orange the ligand N-benzyl-2-chloroacetamide; in green the acid/base catalytic (H224), the oxyanion 1st term (Q106) and 2nd term (T40). Image generated by PyMOL software (http://pymol.org).

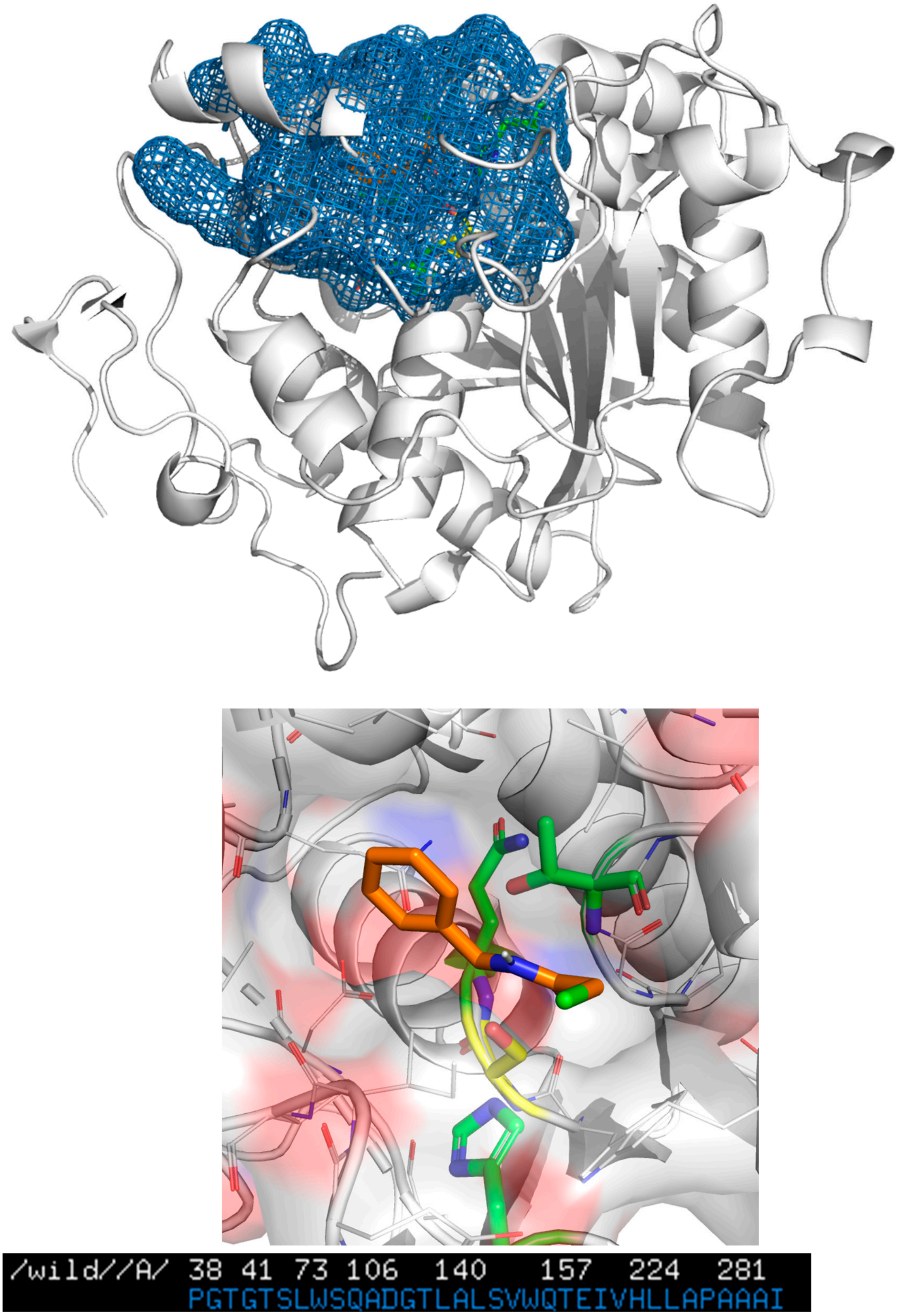

References

Akram,F. *et al.* (2016) Cloning with kinetic and thermodynamic insight of a novel hyperthermostable β-glucosidase from Thermotoga naphthophila RKU-10T with excellent glucose tolerance. *J. Mol. Catal. B Enzym.*, **124**, 92–104.

Bai,A. *et al.* (2013) A novel thermophilic β-glucosidase from Caldicellulosiruptor bescii: Characterization and its synergistic catalysis with other cellulases. *J. Mol. Catal. B Enzym.*, **85–86**, 248–256.

Benoliel,B. *et al.* (2010) Expression of a Glucose-tolerant β-glucosidase from Humicola grisea var. thermoidea in Saccharomyces cerevisiae. *Appl. Biochem. Biotechnol.*, **160**, 2036–2044.

Breves,R. *et al.* (1997) Genes encoding two different beta-glucosidases of Thermoanaerobacter brockii are clustered in a common operon. *Appl. Environ. Microbiol.*, **63**, 3902–3910.

Cao,L. *et al.* (2015) Engineering a novel glucose-tolerant β-glucosidase as supplementation to enhance the hydrolysis of sugarcane bagasse at high glucose concentration. *Biotechnol. Biofuels*, **8**.

Chamoli,S. *et al.* (2016) Secretory expression, characterization and docking study of glucose-tolerant β-glucosidase from B. subtilis. *Int. J. Biol. Macromol.*, **85**, 425–433.

Cota,J. *et al.* (2015) Comparative analysis of three hyperthermophilic GH1 and GH3 family members with industrial potential. *New Biotechnol.*, **32**, 13–20.

Crespim,E. *et al.* (2016) A novel cold-adapted and glucose-tolerant GH1 β-glucosidase from Exiguobacterium antarcticum B7. *Int. J. Biol. Macromol.*, **82**, 375–380.

de Giuseppe,P.O. *et al.* (2014) Structural basis for glucose tolerance in GH1 β-glucosidases. *Acta Crystallogr. D Biol. Crystallogr.*, **70**, 1631–1639.

Gumerov,V.M. *et al.* (2015) A Novel Highly Thermostable Multifunctional Beta-Glycosidase from Crenarchaeon Acidilobus saccharovorans, A Novel Highly Thermostable Multifunctional Beta-Glycosidase from Crenarchaeon Acidilobus saccharovorans. *Archaea Archaea*, **2015**, **2015**, e978632.

Guo,B. *et al.* (2016) Improvements in Glucose Sensitivity and Stability of Trichoderma reesei β-Glucosidase Using Site-Directed Mutagenesis. *PLOS ONE*, **11**, e0147301.

Huang,Y. *et al.* (2014) Identification of a β-glucosidase from the Mucor circinelloides genome by peptide pattern recognition. *Enzyme Microb. Technol.*, **67**, 47–52.

Jabbour,D. *et al.* (2012) A novel thermostable and glucose-tolerant β-glucosidase from Fervidobacterium islandicum. *Appl. Microbiol. Biotechnol.*, **93**, 1947–1956.

Lu,J. *et al.* (2013) Expression and characterization of a novel highly glucose-tolerant β-glucosidase from a soil metagenome. *Acta Biochim. Biophys. Sin.*, **45**, 664–673.

Meleiro,L.P. *et al.* (2015) A Neurospora crassa ß-glucosidase with potential for lignocellulose hydrolysis shows strong glucose tolerance and stimulation by glucose and xylose. *J. Mol. Catal. B Enzym.*, **122**, 131–140.

Pei,J. *et al.* (2012) Thermoanaerobacterium thermosaccharolyticum β-glucosidase: a glucose-tolerant enzyme with high specific activity for cellobiose. *Biotechnol Biofuels*, **5**, 1–10.

Ramani,G. *et al.* (2015) Molecular cloning and expression of thermostable glucose-tolerant β-glucosidase of Penicillium funiculosum NCL1 in Pichia pastoris and its characterization. *J. Ind. Microbiol. Biotechnol.*, **42**, 553–565.

Schröder,C. *et al.* (2014) Characterization of a heat-active archaeal β-glucosidase from a hydrothermal spring metagenome. *Enzyme Microb. Technol.*, **57**, 48–54.

Uchima,C.A. *et al.* (2011) Heterologous expression and characterization of a glucose-stimulated β-glucosidase from the termite Neotermes koshunensis in Aspergillus oryzae. *Appl. Microbiol. Biotechnol.*, **89**, 1761–1771.

Uchima,C.A. *et al.* (2012) Heterologous Expression in Pichia pastoris and Characterization of an Endogenous Thermostable and High-Glucose-Tolerant β-Glucosidase from the Termite Nasutitermes takasagoensis. *Appl. Environ. Microbiol.*, **78**, 4288–4293.

Uchiyama,T. *et al.* (2015) Glucose-tolerant β-glucosidase retrieved from a Kusaya gravy metagenome. *Front. Microbiol.*, **6**.

Yang,F. *et al.* (2015) Overexpression and characterization of a glucose-tolerant β-glucosidase from T. aotearoense with high specific activity for cellobiose. *Appl. Microbiol. Biotechnol.*, **99**, 8903–8915.

Yang,Y. *et al.* (2015) A mechanism of glucose tolerance and stimulation of GH1 β-glucosidases. *Sci. Rep.*, **5**, 17296.
